# Organic phosphorescent nanoscintillator for low-dose X-ray-induced photodynamic therapy

**DOI:** 10.1038/s41467-022-32054-0

**Published:** 2022-08-30

**Authors:** Xiao Wang, Wenjing Sun, Huifang Shi, Huili Ma, Guowei Niu, Yuxin Li, Jiahuan Zhi, Xiaokang Yao, Zhicheng Song, Lei Chen, Shi Li, Guohui Yang, Zixing Zhou, Yixiao He, Shuli Qu, Min Wu, Zhu Zhao, Chengzhu Yin, Chongyang Lin, Jia Gao, Qiuying Li, Xu Zhen, Lin Li, Xiaoyuan Chen, Xiaogang Liu, Zhongfu An, Hongmin Chen, Wei Huang

**Affiliations:** 1 Key Laboratory of Flexible Electronics & Institute of Advanced Materials, Nanjing Tech University, Nanjing, 211800 China; 2 State Key Laboratory of Molecular Vaccinology and Molecular Diagnostics & Center for Molecular Imaging and Translational Medicine, School of Public Health, Xiamen University, Xiamen, 361102 China; 3 ZJU-Hangzhou Global Scientific and Technological Innovation Center, Hangzhou, China; 4 MOE Key Laboratory of High Performance Polymer Materials and Technology, Department of Polymer Science & Engineering, College of Chemistry & Chemical Engineering, and Jiangsu Key Laboratory for Nanotechnology, Nanjing University, Nanjing, 210093 China; 5 The Institute of Flexible Electronics (IFE, Future Technologies), Xiamen University, Xiamen, 361005 Fujian China; 6 Departments of Diagnostic Radiology, Surgery, Chemical and Biomolecular Engineering, and Biomedical Engineering, Clinical Imaging Research Centre, Yong Loo Lin School of Medicine and Faculty of Engineering, National University of Singapore, 117597 Singapore, Singapore; 7 Department of Chemistry, National University of Singapore, 117597 Singapore, Singapore; 8 Frontiers Science Center for Flexible Electronics, MIIT Key Laboratory of Flexible Electronics, Northwestern Polytechnical University, Xi’an, 710072 China

**Keywords:** Cancer therapy, Therapeutics, Targeted therapies

## Abstract

X-ray-induced photodynamic therapy utilizes penetrating X-rays to activate reactive oxygen species in deep tissues for cancer treatment, which combines the advantages of photodynamic therapy and radiotherapy. Conventional therapy usually requires heavy-metal-containing inorganic scintillators and organic photosensitizers to generate singlet oxygen. Here, we report a more convenient strategy for X-ray-induced photodynamic therapy based on a class of organic phosphorescence nanoscintillators, that act in a dual capacity as scintillators and photosensitizers. The resulting low dose of 0.4 Gy and negligible adverse effects demonstrate the great potential for the treatment of deep tumours. These findings provide an optional route that leverages the optical properties of purely organic scintillators for deep-tissue photodynamic therapy. Furthermore, these organic nanoscintillators offer an opportunity to expand applications in the fields of biomaterials and nanobiotechnology.

## Abstract (web summary)

Conventional X-ray-induced photodynamic therapy usually requires heavy-metal-containing inorganic scintillators and organic photosensitizers to generate singlet oxygen. Here, the authors develop an organic phosphorescent nanoscintillator for low-dose X-ray-induced photodynamic therapy of deep tumours.

## Introduction

Photodynamic therapy (PDT), a less invasive strategy for clinical cancer treatment^1–6^, has displayed great potential in oncotherapy^7–9^. In general, three critical constituents play a role in PDT: light, oxygen and photosensitizers. That is, with the help of triplet photosensitizers, cytotoxic reactive oxygen species, in most cases, singlet oxygen (^1^O_2_)^8^, are generated upon appropriate irradiation. In this way, apoptosis or necrosis of malignant cells occurs. A number of efficient triplet photosensitizers excited by visible or ultraviolet light have been developed for PDT^10^. However, owing to the limited penetration depth of visible and ultraviolet light into tissues (<1 cm) ^7,11^, PDT loses its effectiveness in the treatment of deep tumours. Because X-rays can simultaneously penetrate deep tissue^7^ and destroy the DNA^12^ of cancer cells, X-rays have recently been widely adopted in photodynamic therapy. Conventional X-ray-induced photodynamic therapy (X-PDT) uses scintillators to convert X-rays into visible light and then excite nearby photosensitizers via energy transfer (ET)^13–15^. The complicated two-step energy transfer inevitably leads to energy loss by X-ray irradiation^16^, which in turn reduces the performance of X-PDT. To effectively improve energy utilization, the previous scintillating energy transfer strategy was improved by using heavy-metal-containing scintillators such as SrAl_2_O_4_:Eu and LaF_3_:Tb (Supplementary Table 1 and Supplementary Fig. 1). The combination of scintillation and energy transfer processes provides an alternative to improve the performance of X-PDT.

**Fig. 1:**
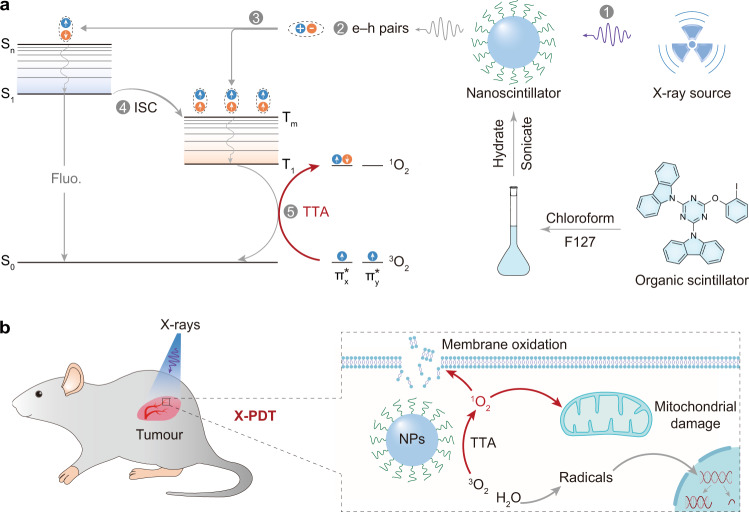
Schematic representation of X-PDT based on organic phosphorescent nanoscintillators. **a** Process to prepare organic nanoparticles in aqueous solution and subsequent ^1^O_2_ generation under X-ray irradiation. Specifically, an organic scintillator 9,9’-(6-iodophenoxy-1,3,5-triazine-2,4-diyl)bis(9H-carbazole) (ITC) and PEG-*b*-PPG-*b*-PEG (F127) were dissolved in chloroform. The resultant solution was evaporated and dissolved in aqueous solution accompanied by intense sonication to obtain organic nanoparticles. Under X-ray irradiation, electrons (orange circles) are mainly ejected from the inner shell of heavy atoms in ITC (step 1), generating massive electron-hole pairs (step 2). The following charge recombination produces singlet and triplet excitons, in a certain ratio. With the assistance of enhanced intersystem crossing (ISC) owing to strong spin-orbit coupling (step 4), a large amount of triplet excitons is produced. The resultant triplet excitons excite ^3^O_2_ to yield ^1^O_2_ by triplet-triplet annihilation (TTA, step 5). **b** Illustration of the mechanism in the treatment of deep-seated tumours in vivo. The local ^3^O_2_ molecules absorb energy to produce massive ^1^O_2_. The resultant ^1^O_2_ species lead to membrane oxidation and mitochondrial damage, which together effectively kill cancer cells.

The key process for PDT is the conversion of ^3^O_2_ to ^1^O_2_ by triplet-triplet annihilation, which requires the participation of triplet photosensitizers (Fig. 1a). In addition, triplet excitons with long lifetimes in photosensitizers are beneficial for the transfer of energy to ^3^O_2_^17^. In view of the long-lived triplet excitons in purely organic luminogens^18–34^, phosphorescent materials can be made useful after improving their X-ray absorption. Modulation of halogen atoms with large atomic number *Z* (e.g., bromine and iodine) can improve the X-ray absorption ability^35–38^, because the attenuation coefficient (*μ*) is proportional to the fourth power of the atomic number (*μ*$\,\propto {Z}^{4}$). Meanwhile, heavy halogen, oxygen, and nitrogen atoms benefit intersystem crossing (ISC) to populate the triplet excitons^39^. After X-ray irradiation, triplet-triplet annihilation (TTA) generates a large amount of ^1^O_2_ for X-PDT with membrane oxidation and mitochondrial damage (Fig. 1b). Therefore, X-PDT can be successfully performed using such efficient organic phosphorescent nanoscintillators. Moreover, benefiting from intersystem crossing (ISC), we had demonstrated that such scintillators can produce more than 75% triplet excitons for ^1^O_2_ generation by X-ray irradiation^40^.

In this work, we employ a purely organic phosphorescent nanoscintillator to realize the X-PDT. This strategy shows efficient energy transfer processes, empowering the phosphorescent nanoscintillator with the ability of producing massive singlet oxygen upon X-ray irradiation. Benefiting from the singlet oxygen triggered PDT and tissue penetration capability of X-rays, we successfully accomplish X-ray triggered dynamic therapy for a combined radio-dynamic therapy of deep tissue tumour under low-dose X-ray irradiation (0.4 Gy). This finding demonstrates great potential of organic phosphorescent nanoscintillator X-PDT in bioelectronics.

## Results

### Synthesis and characterization of nanoscintillator

**Fig. 2:**
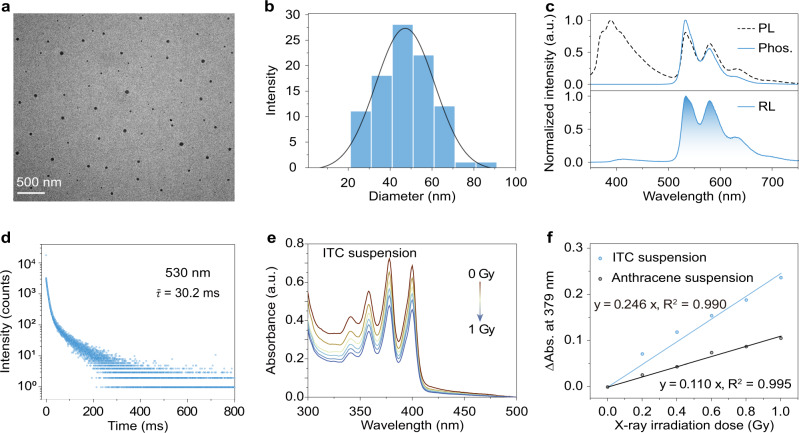
Characterization and photophysical properties of organic phosphorescent nanoscintillators in aqueous solution. **a** TEM image of the prepared nanoparticles (scale bar: 500 nm). **b** Size distribution of nanoparticles obtained by TEM. **c** Steady-state photoluminescence (PL, dashed line) and time-gated phosphorescence (Phos., solid line) spectra of the nanoparticles in solution, respectively (top). And the radioluminescence (RL) spectrum of the nanoparticles in solid state (bottom). **d** Time-resolved decay profile of emission wavelength at 530 nm of the nanoparticles under excitation at 340 nm. **e** Absorption spectra of the aqueous ITC suspension in the presence of anthracene-9,10-diyl-bismethylmalonate (ADMA) under different X-ray irradiation doses (X-ray dose rate: 2.8 mGy/s, time interval: 72 seconds). **f** Dose dependence of the absorbance variation (monitored at 379 nm) of the mixture of ITC (or anthracene) suspension and ADMA in water.

To test this hypothesis, we herein selected 9,9’-(6-iodophenoxy-1,3,5-triazine-2,4-diyl)bis(9H-carbazole) (ITC) as a model scintillator. Apart from the iodine atom, oxygen and nitrogen atoms were also incorporated into the ITC molecule to enhance n–π* transitions, which can further facilitate the ISC process to boost triplet excitons^41^. The chemical structure of ITC was demonstrated by NMR spectroscopy and elemental analysis (Supplementary Section 1 and Supplementary Figs. 2 and 3). Furthermore, we have prepared water-soluble organic nanoscintillators via a top-down approach with an amphiphilic triblock copolymer PEG-*b*-PPG-*b*-PEG (F127). The morphology of the resulting nanoscintillators was investigated by transmission electron microscopy (TEM). As shown in Fig. 2a, the scintillator nanoparticles show spherical morphology with monodisperse sizes of 47.2 ± 0.8 nm (Fig. 2b). In addition, the zeta potential of the as-prepared scintillator nanoparticles is −1.0 mV. These data indicate the good stability in water and uniform morphology of the resulting nanoscintillator.

### Photophysical properties and ^1^O_2_ generation in aqueous solution

We then investigated the photophysical properties of the nanoscintillator in aqueous solution. Under the excitation of ultraviolet light, the nanoparticles show dual emission with a main fluorescence peak at 390 nm and a maximum phosphorescence peak at 530 nm (Fig. 2c). The phosphorescence characteristics of the lower energy emission were confirmed by time-gated photoluminescence (phos.) spectroscopy and time-resolved luminescence decay (Fig. 2c, d). Based on the phosphorescence nature of the scintillator nanoparticles following X-ray irradiation (Fig. 2c), we studied their ability to generate ^1^O_2_. Specifically, we prepared a mixed aqueous suspension of the nanoscintillator and anthracene-9,10-diyl-bismethylmalonate (ADMA, a chemical trap for ^1^O_2_). The resultant mixed suspension was irradiated with X-rays (Fig. 2e), leading to the degradation of ADMA. With prolonged illumination, the representative absorption peaks (360, 379, and 401 nm) of ADMA in aqueous suspension gradually decreased, which signifies the generation of ^1^O_2_ at a low X-ray dose. Compared with the anthracene, a typical organic scintillator to evaluate ^1^O_2_ generation, the ITC suspension showed a 2-fold higher dependence of ^1^O_2_ generation on X-ray dose (Fig. 2f). Furthermore, we also employed a commercial photosensitizer, chlorin E6 (Ce6)^42^, to demonstrate its ^1^O_2_ production capacity upon activation by visible light or X-rays (Supplementary Fig. 4).

### In vitro evaluation of X-ray-induced photodynamic therapy

**Fig. 3:**
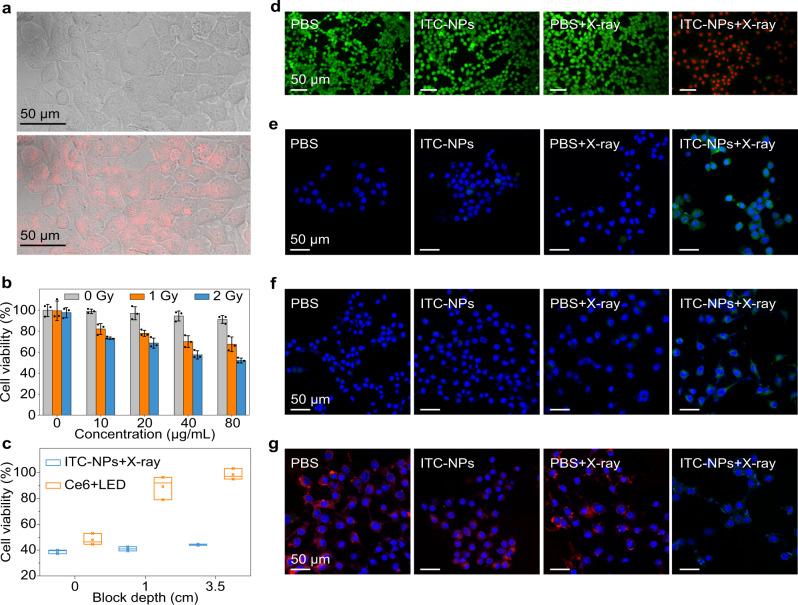
Evaluation of organic phosphorescent nanoscintillators for X-PDT in vitro. **a** Confocal microscopic images of 4T1 cells after incubation with ITC nanoparticles (ITC-NPs). The red colour (bottom) is from the phosphorescence of the ITC-NPs, indicating effective cell uptake, as it appears in the same position as the bright-field images of the 4T1 cells (top). **b** Viability of 4T1 cells incubated with different concentrations of ITC-NPs. Note that each group was treated parallelly with or without X-ray irradiation (1 or 2 Gy). The statistical data are expressed as mean values ± S.D. (*n* = 3 independent experiments). Note that the total dose that medium and cells irradiated is 1-2 Gy. **c** Chicken breast block depth dependence of viability of 4T1 cells incubated with ITC-NPs (X-ray: 2 Gy) or Ce6 (LED light: 670 nm, 300 mW/cm^2^, 90 J/cm^2^, 300 s). Median and interquartile ranges are presented for the box plot (*n* = 3 independent experiments). **d** Calcein-AM/PI co-staining assay of 4T1 cells after treatment with PBS, PBS + X-rays, ITC-NPs, and ITC-NPs+X-rays (X-ray irradiation: 2 Gy) (Green: live cells; Red: dead cells). **e** Confocal laser scanning microscope (CLSM) images of 4T1 cells stained with SOSG after incubation with PBS or ITC-NPs. Two treatment groups were subjected to X-ray irradiation (2 Gy). **f** CLSM images of lipoperoxides in 4T1 cells after incubation with PBS or ITC-NPs with or without X-ray irradiation (2 Gy). Green fluorescence indicates lipid ROS formation after staining with BODIPY-C11. **g** CLSM images of the changes in the mitochondrial membrane potential of 4T1 cells irradiated with or without X-rays (2 Gy). Red fluorescence indicates positive membrane potential, whereas green fluorescence indicates decreased membrane potential.

Benefiting from the remarkable ability of scintillator nanoparticles to generate ^1^O_2_, we performed MTT (4-nitrophenyl chloroformate 3-(4,5-dimethylthiazol-2-yl)−2,5-diphenyltetrazolium bromide) assay to evaluate X-PDT in vitro using 4T1 mouse breast cancer cells. After incubation with scintillator nanoparticles (80 μg/mL) for 24 h, 4T1 cells exhibited an intense, clear, and homogeneous photoluminescence signal in the cytosol (Fig. 3a), demonstrating the endocytosis process of the nanoscintillator by 4T1 cells. Then, the X-PDT effect of the scintillator nanoparticles on 4T1 cells was conducted. Scintillator nanoparticles without X-ray irradiation, even at a high concentration of 80 μg/mL, have no obvious cytotoxicity (Fig. 3b). However, once X-ray (1.0 Gy) was applied, the dramatically decreased cell viability was accompanied by an increase in the concentration of scintillator nanoparticles. This situation intensified when the X-ray dose was increased to 2.0 Gy, indicating the preliminary success of X-PDT. In contrast, the blank F127 micelles displayed weak cytotoxicity on 4T1 tumor cells (Supplementary Fig. 5), which further demonstrates the importance of the nanoscintillator in X-PDT. It is worth noting that these conclusions are in good agreement with the live/dead-cell staining assay. This kit contains calcein-AM and propidium iodide (PI), which stain viable cells with green fluorescence and dead cells with red fluorescence. Under the same conditions, only the X-PDT group can efficiently induce cancer cell death (Fig. 3d). Compared with traditional PDT, the main advantage of deep-tissue penetration during X-PDT was also evaluated (Fig. 3c). Impressively, X-PDT achieved a great therapeutic effect even when a 3.5 cm chicken breast was used as a block (Supplementary Fig. 6), while only 1 cm chicken breast weakened the efficacy of traditional PDT.

In the following set of experiments, we investigated the mechanism of X-PDT on 4T1 cells. First, we examined intracellular ^1^O_2_ generation during X-PDT. Singlet oxygen sensor green (SOSG) was selected as a probe for ^1^O_2_ because it shows intense green fluorescence after the cycloaddition reaction with ^1^O_2_. Intense green fluorescence was detected only in 4T1 cells treated with the scintillator nanoparticles by X-ray irradiation, while phosphate-buffered saline (PBS) groups, and the control group without X-ray irradiation showed no green emission (Fig. 3e, Supplementary Fig. 7). This suggests that ^1^O_2_ can be generated intracellularly when the scintillator nanoparticles were irradiated with X-rays. To gain a deeper insight into the pharmacology of ^1^O_2_, we then inspected the oxidative degradation of unsaturated lipids and surface proteins in 4T1 cell membranes using a lipid peroxidation assay as described in the previous reports^11^. The results clearly showed that the X-PDT group exhibited a higher level of lipid peroxidation than the control groups, which was due to the apparent green fluorescence when stained with BODIPY-C11 (Fig. 3f and Supplementary Fig. 8). Furthermore, the situation of mitochondria in 4T1 cells was also studied. As can be seen, the mitochondrial membrane potential of 4T1 cells after incubation with nanoscintillators and subsequent X-ray irradiation was lower than the control groups (Fig. 3g, Supplementary Fig. 9), implying that ^1^O_2_ causes mitochondrial dysfunction in 4T1 cells. Moreover, the flow cytometry results matched well with the in vitro comparison between different groups (Supplementary Fig. 10). Taking together, we clarified the X-PDT mechanism of the organic scintillator nanoparticles. In brief, with the aid of the large stopping power and the enhanced spin-orbit coupling effect caused by the iodine atoms, the nanoscintillator revealed good performance in producing triplet excitons upon X-ray irradiation, which gives it the ability to generate ^1^O_2_. The resultant ^1^O_2_ then effectively damages cell membranes and mitochondria of 4T1 cells to achieve cancer treatment. The radiotherapeutic effect was then tested using single-cell electrophoresis, the comet assay. Compared with the PBS + X-ray group, the X-PDT group showed similarly frequent DNA strand breaks of 4T1 cells because of the comet-like appearance (Supplementary Fig. 11), demonstrating the effect of radiotherapy in the X-PDT group. The proliferative potential of cells after X-PDT was tested by clonogenic assay (Supplementary Fig. 12). At the same X-ray dose, the X-PDT group showed weaker proliferation than the PBS + X-ray group, which proves that X-PDT treatment effectively inhibits the colony-forming ability. ITC-NPs showed radiosensitization with a radiation enhancement factor of 1.53 at 10% survival fractions. These results imply that our X-PDT system exhibits a weak radiosensitizing effect, but it is mainly due to the PDT effect.

Before the bioapplications of nanoscintillators, we evaluated the biosafety and long-term toxicity in Balb/c mice by a dose-escalation study. Balb/c mice were intravenously injected with PBS or nanoscintillators (50 mg kg^−1^) daily for 3 days. Blood samples were collected for serum chemistry and haematological analyses on day 1, day 15 and day 30 after treatment. Treatment with nanoscintillators for three consecutive days caused no significant changes in mice’s whole blood (Supplementary Fig. 13) and serum chemistry, including liver and renal functions and myocardial enzymogram (Supplementary Fig. 14). In addition, no significant weight loss was observed (Supplementary Fig. 15). Therefore, the nanoscintillators can be considered to have low acute and subacute toxicity in vivo.

### In vivo evaluation of X-ray-induced photodynamic therapy

**Fig. 4:**
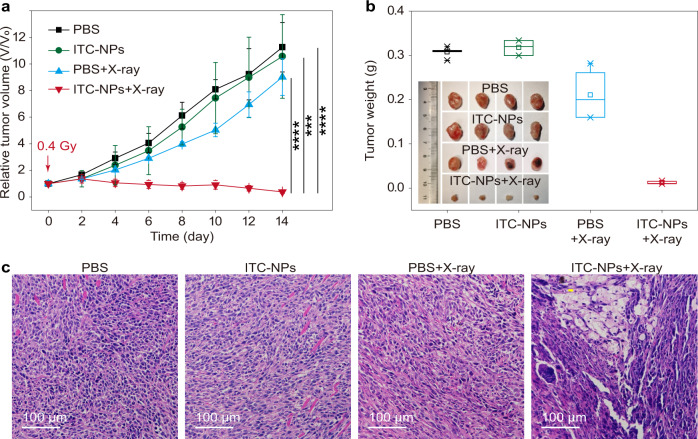
Assessment of organic phosphorescent nanoscintillator for X-PDT in vivo after intravenous injection. **a** Tumour growth curves of 4T1 tumours with different treatments (****P < 0.0001, ***P = 0.0003). On day 0, 0.4 Gy X-ray irradiation was given without further treatment. The statistical data are expressed as mean values ± S.D. (*n* = 5 biologically independent animals). Statistical significance was assessed via unpaired two-sided Student *t*-test. **b** Tumour weights after different treatments for 14 days. The inset shows the resulting tumour photographs. Median and interquartile ranges are presented for the box plot (*n* = 5 biologically independent samples). **c** H&E-stained images of tumour slices after various treatments.

Having seen the efficient anti-tumour effect of X-ray-based PDT in vitro, we evaluated the accumulation of nanoscintillators in 4T1 tumours. We introduced a fluorescent dye Cy5.5-COOH into the nanoscintillator to make it detectable by an in vivo imaging system (Supplementary Fig. 16a). An increase in fluorescence intensity (λ_ex_/λ_em_ = 645 nm/Cy5.5) was observed in the tumour region over time. The plateaus at 24 h post-injection prove effective accumulation in the tumours (Supplementary Fig. 16b). Furthermore, the biodistribution and clearance behaviour of nanoscintillators in mice with 4T1 tumours suggest that nanoscintillators had a long circulation half-life (187.8 min, Supplementary Fig. 17). As a result, nanoscintillators accumulated to a high extent in the 4T1 tumors (Supplementary Fig. 18). Encouraged by these results, we subsequently evaluated the in vivo antitumour efficacy. First, an initial dose of 0.4 Gy was used, and the scintillator nanoparticles were injected intratumorally into tumour-bearing mice. The resultant good efficacy (Supplementary Figs. 19–23) encouraged us to investigate intravenous injection therapy. When 4T1 tumours reached a volume of ~80 mm^3^, the mice were randomly divided into four groups: PBS only, ITC-NPs only (0.4 mg mL^−1^, 0.2 mL), PBS + X-ray, and ITC-NPs+X-ray (0.4 mg mL^−1^, 0.2 mL, namely X-PDT). Compared with tumours treated with PBS only and ITC-NPs only, the PBS + X-ray groups exhibited delayed tumour growth, demonstrating the limited efficacy of radiotherapy (RT). In contrast, X-PDT treatment was effective in suppressing tumour growth at an extremely low X-ray dose (0.4 Gy) (Fig. 4a). As evidence, we supplied the photographs of tumour-bearing mice on days 0, 6 and 14 (Supplementary Fig. 24). In addition, the weights and images of tumours treated with different approaches were provided at the endpoint (Fig. 4b), also highlighting the curative effect of X-PDT. We also utilized hematoxylin & eosin (H&E) staining of tumour slices to expose the damaged structures in the tumours, aiming to show the visibly reduced cancer cell density in the X-PDT group. X-PDT triggered late-stage cancer cell apoptosis, including pyknosis, karyolysis and others, which outperformed all other treatment groups (Fig. 4c).

We next evaluated the biosafety of the X-PDT procedure. Throughout the treatment, the body weight of the mice remained relatively stable in all groups (Supplementary Fig. 25). Meanwhile, the major organs such as heart, liver, spleen, lungs and kidneys showed no signs of systemic toxicity, as evidenced by H&E staining (Supplementary Fig. 26). Moreover, no significant abnormalities were observed in the serum chemistry of the mice and the complete blood analysis (Supplementary Figs. 27 and 28). These results proved that X-PDT has good efficacy in treating deep tumours by combining the advantages of PDT and RT.

In summary, we have reported a low-dose X-ray-induced photodynamic therapy based on purely organic phosphorescent nanoscintillators. By combining scintillation and energy transfer processes, scintillator nanoparticles are endowed with the ability to generate massive singlet oxygen upon X-ray irradiation, which improves the performance of X-PDT. In particular, scintillator nanoparticles validate good deep tumour therapy in vivo under low-dose X-ray irradiation (0.4 Gy), accompanied by negligible adverse effects on normal organs. This strategy combines the advantages of PDT and RT to advance the development of cancer therapy in deep tissue. Our results also provide a promising alternative to the current X-PDT based on heavy-metal-containing agents.

## Methods

### Reagents and solvents

Unless otherwise noted, reagents and solvents were purchased directly from chemical sources and used without further treatment. Organic reactions proceeded in a nitrogen atmosphere using standard air-free Schlenk techniques. Tetrahydrofuran (THF) was dried and distilled before using, sodium (Na) and benzophenone were added as colour indicators. All products were purified by flash column chromatography, followed by further crystallisation techniques such as slow cooling to obtain highly purified products.

### Preparation of nanoscintillators

PEG-*b*-PPG-*b*-PEG (F127, 10 mg) and ITC (1 mg) were dissolved in 1 mL chloroform and then evaporated to form a thin film. The thin film was hydrated in 3 mL of D.I. water under ice-bath and sonicated vigorously at 10 min intervals, followed by filtration through a 0.22 μm PVDF syringe-driven filter. After washing three times with deionized (D.I.) water to remove unencapsulated molecules by ultrafiltration, the formed nanoparticles were suspended in phosphate buffer saline (PBS) for further use. Subsequently, the resulting nanoparticle solution was heated to remove the water, which produced a white solid (named ITC-F127). Then, 100 μL of dimethyl sulfoxide (DMSO), 900 μL of sterile water, and 1 mg ITC-F127 solid were mixed and sonicated for 5 min, generating a mother solution with a concentration of 1 mg/mL. Finally, we diluted the mother solution by adding various ratios of PBS.

### Preparation of the nanosystem for fluorescent imaging

PEG-*b*-PPG-*b*-PEG (F127, 10 mg), ITC (0.5 mg) and Cy5.5-COOH (CAS No. 1144107-80-1, 0.5 mg) were dissolved in 1 mL of chloroform and then evaporated to form a thin film. Following the same procedure to prepare the nanoscintillators, the nanosystem for fluorescent imaging study was conducted.

### ^1^O_2_ generation in suspension following X-ray irradiation

To demonstrate the generation of ^1^O_2_, ADMA was used as a detection probe due to its variation in the absorption spectrum. Considering the encapsulation efficiency of F127 to ITC, anthracene and Ce6 is different, it is difficult to compare these nanoparticles if we need to strictly keep the effective mass of agents equal. Therefore, we directly compared the corresponding properties of ITC and anthracene (or Ce6) in water. Specifically, ITC was suspended in water (370 μg/mL) in the presence of 150 μg/mL of ADMA. By replacing ITC with anthracene (or Ce6), a parallel experiment was employed as a control group. These two groups were irradiated with X-rays at a dose rate of 2.8 mGy/s. With increasing irradiation time (interval: 72 seconds), the UV-Vis absorption spectra were measured. Notably, the photochemistry will be less in suspension than it would have been in solution under the same conditions.

### Cell uptake

4T1 cells were incubated with scintillator nanoparticles (80 μg/mL). After 24 h, cells were washed with PBS and replenished with fresh medium. Fluorescence images were acquired using an Olympus FV1200 laser scanning confocal microscope.

### Cytotoxicity

The 4T1 cells were cultured in DMEM medium containing 10% FBS in a humidified atmosphere with 5% carbon dioxide at 37 °C for 24 h. The viability of 4T1 cells was studied using the standard MTT assay. Cells were seeded on 96-well plates (10^4^ cells per well) and incubated for 24 h prior to the next experiments. Scintillator nanoparticles at different concentrations (0, 10, 20, 40 and 80 μg/mL) were added to the medium and incubated with the cells for 24 h. MTT assay was then conducted. To study the viability of cells as a function of block depth, we covered different chicken breast pieces to imitate various tissue depths.

### In vitro X-PDT assay

The 4T1 cells were incubated with scintillator nanoparticles (80 μg/mL) for 24 h and washed three times with PBS. X-ray irradiation was carried out at a minimum dose of 1.0 Gy (50 kV). After incubation for another 24 h, the medium was abandoned and cells were washed three times with PBS. The viability of the cells was determined using the MTT assay. The 4T1 cells were incubated with PBS or scintillator nanoparticles (80 μg/mL). After 24 h, the cells were washed with PBS and replenished with fresh medium. The X-ray irradiation group received a dose of 2.0 Gy of X-ray irradiation (50 kV). After a further incubation of 24 h, the viability of cells was determined using the MTT assay. To observe the condition of 4T1 cells in vitro, they were seeded in 6-well petri-dishes and cultured for 24 h. After different treatments with PBS, PBS + X-rays, scintillator nanoparticles (80 μg/mL), or scintillator nanoparticles (80 μg/mL) + X-rays, cells were stained with calcein-AM/PI for 30 min at 37 °C and washed three times with PBS. All images were acquired using an Olympus FV1200 laser scanning confocal microscope.

### ^1^O_2_ generation in cells following X-ray irradiation

To monitor intracellular ^1^O_2_ generation, approximately 200,000 4T1 cells were seeded in a glass-bottomed cell culture dish (diameter: 10 mm) and incubated for 24 h. Subsequently, cells were replenished with a fresh medium containing 80 μg/mL scintillator nanoparticles. After incubation for 8 h and washing with PBS, 5 μM of SOSG were added and incubated for 30 minutes. A control group was treated with PBS only. After washing with PBS, cells were irradiated with X-rays at a dose of 2 Gy (50 kV). Luminescence images were taken using an Olympus FV1200 laser scanning confocal microscope.

### Lipid peroxidation measurement

200,000 4T1 cells were seeded in a glass-bottomed cell culture dish (diameter: 10 mm) and incubated for 24 h. Four groups were randomly assigned: blank control group (PBS only), PBS + X-rays, scintillator nanoparticles (80 μg/mL), and scintillator nanoparticles (80 μg/mL) + X-rays. The two treatment groups were irradiated with X-ray (2.0 Gy, 50 kV). Confocal microscopic analysis with BODIPY-C11 staining was then used to evaluate lipid peroxidation.

### Measurement of mitochondrial membrane potential

200,000 4T1 cells were seeded in a glass-bottomed cell culture dish (diameter: 10 mm) and incubated for 24 h. Four groups were randomly assigned: blank control group (PBS only), PBS + X-ray, scintillator nanoparticles (80 μg/mL), and scintillator nanoparticles (80 μg/mL) + X-ray. The two treatment groups were irradiated with X-ray (2.0 Gy, 50 kV). Cells were then tested for changes in mitochondrial membrane potential, based on a JC-1 Mitochondrial Membrane Potential Assay Kit (MedChemexpress Co., Ltd) according to the vendor’s protocol.

### Comet assay

200,000 4T1 cells were seeded in 6-well plates and incubated for 24 h. Four groups were randomly assigned: blank control group (PBS only), PBS + X-rays, scintillator nanoparticles (80 μg/mL), and scintillator nanoparticles (80 μg/mL) + X-rays. The two treatment groups were irradiated with X-ray (2.0 Gy, 50 kV). Cells were then combined with molten LMAgarose (at 37 °C) at a ratio of 1:10 (v/v). 50 μL of the solution was pipetted onto a CometSlide™. The slide was then immersed overnight in a 4 °C lysis solution. 4°C 1× neutral electrophoresis buffer was added to the electrophoresis gel box and the slides were placed in a slide tray. The power supply was set at 21 volts. After 45 min, the slides were gently removed and immersed in DNA precipitation solution for 30 min. They were then placed in 70% ethanol for 30 min at room temperature. The slides were dried and stained in SYBR® safe for 30 min in the dark. Images of the individual cell nuclei were acquired using an inverted fluorescence microscope.

### Clonogenic assay

200,000 4T1 cells were seeded in 6-well plates and co-incubated with PBS or ITC-NPs for 24 h. 0, 2, 4, 6, 8 and 10 Gy of X-ray irradiation were applied. Then, cells were harvested and 1,000 cells were redistributed in 6-well plates. After incubation for 14 days, cells were fixed and stained with 0.5% gentian violet. Colonies containing more than 50 cells were counted and survival fractions were calculated. The dose enhancement factor (1.53) was calculated as the ratio between the radiation doses of PBS + X-rays (9.40 Gy) and ITC-NPs+X-rays (6.15 Gy) at 10% survival fractions.

### Animal experiments

All animal experimental procedures were performed following the guidelines of the Regional Ethics Committee for Animal Experiments and the Care Regulations approved by the Institutional Animal Care and Use Committee of Xiamen University.

### In vivo imaging, biodistribution, and blood clearance evaluation of nanoscintillators with Cy5.5-COOH

Tumour-bearing mice were established by subcutaneously injecting 2 × 10^6^ 4T1 cells. When the tumour size reached 60~80 mm^3^, nanoscintillators with Cy5.5-COOH were injected intravenously. Fluorescent images were conducted at 1, 2, 4, 8, 12 and 24 h post-injection time utilizing an IVIS Lumina II in vivo imaging system. In addition, the ex vivo imaging was conducted. 4T1 tumour-bearing mice were euthanized and dissected at different post-injection times (0.5, 1, 2, 4, 8, 12, 24 and 48 h). The dissected main organs and tumours were analysed using an IVIS Lumina II in vivo imaging system. The blood (ca. 200 µL) was weighed, and then determined by fluorescence intensity, and calculated as the percentage of injected dose per gram of tissue (%ID/g). The in vivo blood circulation half-life of the nanosystem was calculated by a double-component pharmacokinetic model.

### In vivo X-PDT tests

For tumour therapy, 4T1 tumour-bearing mice were randomly divided into four groups: PBS only, ITC only, PBS + X-rays, and ITC + X-rays. The mice were injected with scintillator nanoparticles (200 μL, 2 mg/mL) on the first day (two methods: intratumoral and intravenous injection). X-ray irradiation at a dose of 0.4 Gy (50 kV) was applied to tumour areas at 8 h post-injection, while the rest of the body of the mice was shielded by lead. The body weights and tumour size of each group were recorded every other day during the 14-day treatment. Tumour volume was determined using the equation: Tumor volume = (width^2^ × length)/2. After tumour therapy, all mice were euthanized. The tumours were excised and photographed. The excised tumours and major organs were sliced and then subjected to standard H&E staining for histological analysis.

### Hematological analysis

Hematological analyses of mice were conducted in four groups: PBS, scintillator nanoparticles, PBS + X-rays, and scintillator nanoparticles + X-rays. Mice were treated intravenously with PBS or scintillator nanoparticles (0.4 mg/mL, 200 μL). Blood samples were collected on day 14 after treatment.

### Statistical analysis

All data were presented as mean values ± standard deviation (S.D.). Comparison of data was conducted with an unpaired two-sided Student’s *t*-test using GraphPad Prism 7.00. Differences were considered statistically significant (*P < 0.05, **P < 0.01, ***P < 0.001, and ****P < 0.0001).

### Statistics and reproducibility

Unless specifically mentioned in figure legends, each experiment was repeated at least three times independently with similar results.

### Reporting summary

Further information on research design is available in the Nature Research Reporting Summary linked to this article.

## Supplementary information

Supplementary Information

Reporting Summary

## Data Availability

The imaging data within the paper are available from the corresponding authors upon request. The authors declare that all other data supporting the findings of this study are provided in the Supplementary Information/Source Data file. Source data is available for Figs. 2, 3 and 4 and Supplementary Figs. 1, 4, 5, 12, 13, 14, 15, 16, 17, 20, 22, 23, 25, 27 and 28 in the associated source data file. Source data are provided with this paper.

## Source data

Source Data
