# Supplementary material for: Organic phosphorescent nanoscintillator for low-dose X-ray-induced photodynamic therapy
Source: Nat Commun. 2022 Aug 30;13:5091. doi: 10.1038/s41467-022-32054-0 (PMC9428140; doi:10.1038/s41467-022-32054-0)
Supplement: Supplementary file 1 — Supplementary Information [file 41467_2022_32054_MOESM1_ESM.pdf]

Supplementary Information

Organic phosphorescent nanoscintillator for low-dose X-ray-induced photodynamic therapy

Xiao Wang[1,&], Wenjing Sun[2,3,&], Huifang Shi[1], Huili Ma[1], Guowei Niu[1], Yuxin Li[1], Jiahuan Zhi[1], Xiaokang Yao[1], Zhicheng Song[1], Lei Chen[2], Shi Li[2], Guohui Yang[1], Zixing Zhou[1], Yixiao He[1], Shuli Qu[1], Min Wu[4], Zhu Zhao[1], Chengzhu Yin[1], Chongyang Lin[1], Jia Gao[1], Qiuying Li[1], Xu Zhen[4], Lin Li[1,5], Xiaoyuan Chen[6], Xiaogang Liu[7], Zhongfu An[1,5*], Hongmin Chen[2*] and Wei Huang[1,5,8*]

[1]Key Laboratory of Flexible Electronics & Institute of Advanced Materials, Nanjing Tech University, Nanjing, 211800, China.
[2]State Key Laboratory of Molecular Vaccinology and Molecular Diagnostics & Center for Molecular Imaging and Translational Medicine, School of Public Health, Xiamen University, Xiamen 361102, China.
[3]ZJU-Hangzhou Global Scientific and Technological Innovation Center, Hangzhou, China.
[4]MOE Key Laboratory of High Performance Polymer Materials and Technology, Department of Polymer Science & Engineering, College of Chemistry & Chemical Engineering, and Jiangsu Key Laboratory for Nanotechnology, Nanjing University, Nanjing 210093, China.
[5]The Institute of Flexible Electronics (IFE, Future Technologies), Xiamen University, Xiamen 361005, Fujian, China.
[6]Departments of Diagnostic Radiology, Surgery, Chemical and Biomolecular Engineering, and Biomedical Engineering, Clinical Imaging Research Centre, Yong Loo Lin School of Medicine and Faculty of Engineering, National University of Singapore, 117597, Singapore, Singapore.
[7]Department of Chemistry, National University of Singapore, 117597, Singapore, Singapore.
[8]Frontiers Science Center for Flexible Electronics, MIIT Key Laboratory of Flexible Electronics, Northwestern Polytechnical University, Xi'an 710072, China.
[&] These authors contributed equally to this work.
**e-mail: iamzfan@njtech.edu.cn; hchen@xmu.edu.cn; iamwhuang@njtech.edu.cn*

Contents

I. Investigation of X-PDT. **S2**
II. Experimental section. **S4**
III. Singlet oxygen generation in solution following X-ray irradiation. **S6**
IV. *In vitro* experiments. **S7**
V. Long-term toxicity evaluation. **S11**
VI. *In vivo* fluorescent imaging and clearance behaviours. **S13**
VII. *In vivo* experiments *via* intratumoral injection. **S14**
VIII. *In vivo* experiments *via* intravenous injection. **S17**
IX. References **S21**

I. Investigation of X-PDT.

Supplementary Table 1. The development history and situation of X-PDT.

Scintillator	Photosensitizer	Material type[a]	Dose[b]	Exp. subject[c]	Remark
LaF_3:Tb	MTCP	Metal-containing	N/A	in solution	Ref. 1
TiO_2, ZnS:Ag, CeF_3, CdTe *etc.*	self		1-10 Gy	in cell	Ref. 2
Gd_2O_2S:Tb	photofrin II		N/A	in cell	Ref. 3
Y_2O_3	psoralen		2 Gy	in cell	Ref. 4
Tb_2O_3	porphyrin		14.6 Gy	in solution	Ref. 5
ZnS:Cu,Co	TBrRh123		2 Gy	in cell	Ref. 6
$LiYF_4$:Ce	ZnO		8 Gy	in animal (it)	Ref. 7
LaF_3:Tb	rose bengal		N/A	in solution	Ref. 8
$[M_6Li_8La_6]^n$ complex	self		N/A	in solution	Ref. 9
$SrAl_2O_4$:Eu	MC540		0.5 Gy	in animal (it)	Ref. 10
GdEuC12	hypericin		N/A	in solution	Ref. 11
LaF_3:Tb	rose bengal		N/A	in solution	Ref. 12
$SrAl_2O_4$:Eu@mSiO_2	MC540		5 Gy	in animal (it)	Ref. 13
$[Hf_6O_4(OH)_4(HCO_2)_6]$ SBUs	$Ir[bpyppy)_2]^+$<br>$[Ru(bpy)_3]_2^+$		N/A	in animal (it)	Ref. 14
$LiGa_5O_8$:Cr	2,3-naphthalocyanine		6 Gy	in animal (iv)	Ref. 15
Hf-DBB-Ru	self		N/A	in animal (iv)	Ref. 16
Hf_6 SBUs *etc.*	Fe-TBP		5*0.5 Gy	in animal (it)	Ref. 17
TBP-Hf nMOFs *etc.*	self		0.5 Gy/fraction	in animal (it, iv)	Ref. 18
$LiLuF_4$:Ce	Ag_3PO_4-Pt(IV)		4 Gy	in animal (it)	Ref. 19
$ZnGa_2O_4$:Cr/W	$ZnPcS_4$		0.18 Gy	in animal (iv)	Ref. 20

Copper-cysteamine	self		5 Gy	in animal (iv)	Ref. 21
$Gd_2(WO_4)_3$:Tb	MC540		6 Gy	in animal (it, iv)	Ref. 22
Zn_2SiO_4:Mn	rose bengal		1.5 Gy	in animal (iv)	Ref. 23
AIE-Au	rose bengal		1 Gy	in animal (iv)	Ref. 24
AuNPs and verteporfin	verteporfin	Metal-containing	4 Gy	in animal (it)	Ref. 25
AuNPs and verteporfin	verteporfin		4 Gy	in cell	Ref. 26
Copper-cysteamine	self		5 Gy	in animal (iv)	Ref. 27
Hb@Hf-Ce6	chlorin e6		6 Gy	in animal (iv)	Ref. 28
Hf-AIE	TPE-DAC		8 Gy	in animal (iv)	Ref. 29
SiC/SiO_x	H2TPACPP	Metalloid-containing	2 Gy	in cell	Ref. 30
ITC	self	Purely organic	0.4 Gy	in animal (it, iv)	This work

[a] The material includes scintillator and photosensitizer. [b] N/A, not available. [c] it, intratumoral injection; iv, intravenous injection.

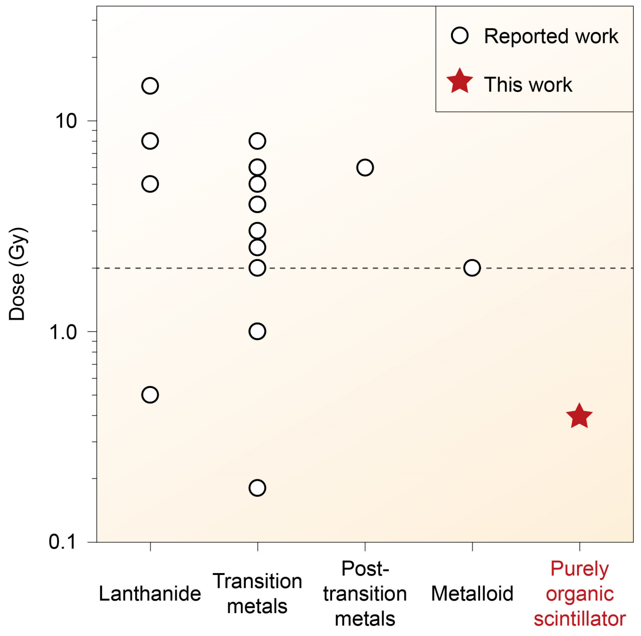

Supplementary Fig. 1 | Distribution showing dose of X-ray and materials type during X-PDT[1-30].

II. Experimental section.

Measurements. ^{1}H and ^{13}C nuclear magnetic resonance spectra were collected using a Bruker Ultra Shield Plus spectrometer (400 MHz). Chemical shifts were calibrated by using tetramethylsilane (TMS) in deuterated solvents as the internal standard. Elemental analysis was accomplished on a Vario EL Cube. Steady-state luminescence and excitation spectra were recorded using Hitachi F-7100 and Edinburgh FLS1000 fluorescence spectrophotometers. The lifetime was obtained on a fluorescence spectrophotometer (Edinburgh FLS1000) equipped with a xenon arc lamp (Xe900), a nanosecond hydrogen flash (nF920), or a microsecond flash (µF900). TEM image was taken on 2100Plus transmission electron microscope (JEOL Ltd., Tokyo, Japan). Zeta potential was measured on a NanoPlus-3 Particle Analyzer (OTSUKA). The fluorescence images of cells were taken on a laser scanning confocal microscopy (Olympus FV1200, Japan).

Synthesis of 9,9'-(6-(2-iodophenoxy)-1,3,5-triazine-2,4-diyl)bis(9H-carbazole) (ITC). This molecule was prepared according to the previously reported literature[S31]. In a nitrogen-filled two-necked flask with 9H-carbazole (5 g, 29.9 mmol), 40 mL dry and degassed THF was slowly injected. Afterward, *n*-butyllithium (20.5 mL, 1.6 mol/L in hexane) was added dropwise at 273 K. The resulting mixture was stirred at room temperature for 2 hours. Following the same procedure, a solution of 2,4,6-trichloro-1,3,5-triazine (2.7 g, 14.9 mmol) in THF (10 mL) was prepared. Subsequently, these two solutions were slowly blended under nitrogen atmosphere at 353 K and stirred for about 8 hours. Once the reaction was completed, the mixture was filtered and washed with cold acetone. Faint yellow powders (9,9'-(6-chloro-1,3,5-triazine-2,4-diyl)bis(9H-carbazole), CzDClT, 3 g, 6.7 mmol) were obtained in 45% yield. To a solution of *o*-iodophenol (2.2 g, 10.1 mmol) and dry THF (15 mL), a freshly prepared solution of sodium hydroxide (0.56 g, 2.0 mol/L in DI water) was slowly added and stirred at 298 K for 1 hour. The mixture was then added into a round-bottomed flask charged with CzDClT (3 g in 50 mL dry THF), and the solution was stirred and refluxed at 350 K for 1 hour. The crude solution was evaporated and extracted with dichloromethane three times. The resulting organic layer was dried using anhydrous sodium sulfate. After the solvent was removed by rotary evaporation, the residue was purified by flash column chromatography to give *o*-ITC (2.9 g, 68%) as a white solid. Before using, the white solid was recrystallized by a mixed solvent (chloroform/ethanol). ^{1}H NMR ($CDCl_3$): δ 8.72 (dd, J = 6.2, 3.0 Hz, 4H), 8.09-8.00 (m, 5H), 7.58 (td, J = 7.8, 1.5 Hz, 1H), 7.47 - 7.35 (m, 9H), 7.22 (td, J = 7.8, 1.5 Hz, 1H). ^{13}C NMR ($CDCl_3$): δ 171.35, 165.63, 153.10, 140.07, 138.86, 130.14, 128.03, 127.08, 126.76, 123.74, 119.74, 118.02, 91.51. Anal. Calculated for $C_{33}H_{20}IN_5O$: C, 62.97; H, 3.20; N, 11.13. Found: C, 63.34; H, 3.29; N, 11.08.

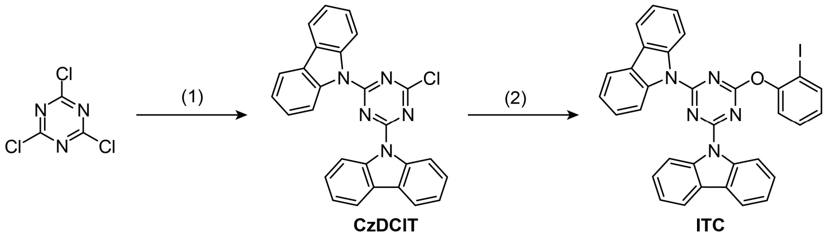

Supplementary Scheme 1. Molecular structure and the synthetic route of ITC molecule. (1) 9H-carbazole, *n*-butyllithium, 303 K, 12 h; (2) *o*-iodophenol, NaOH, 350 K, 1 h.

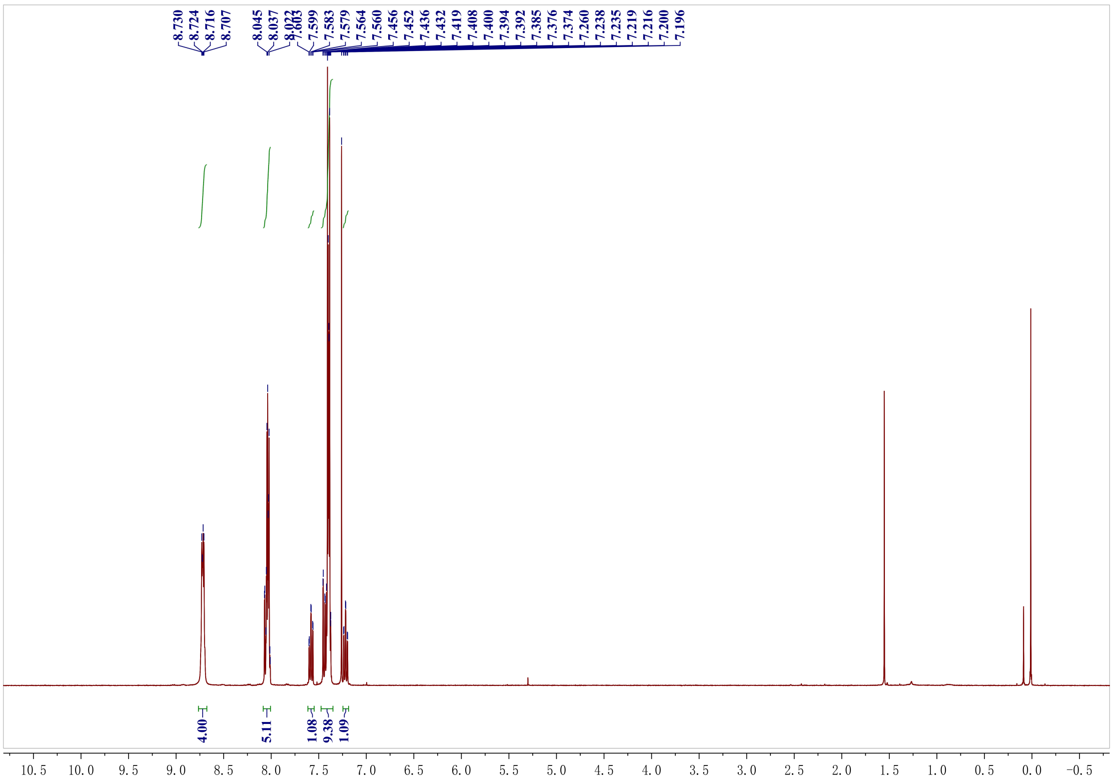

Supplementary Fig. 2 | The ^{1}H NMR spectrum of ITC molecule in $CDCl_3$.

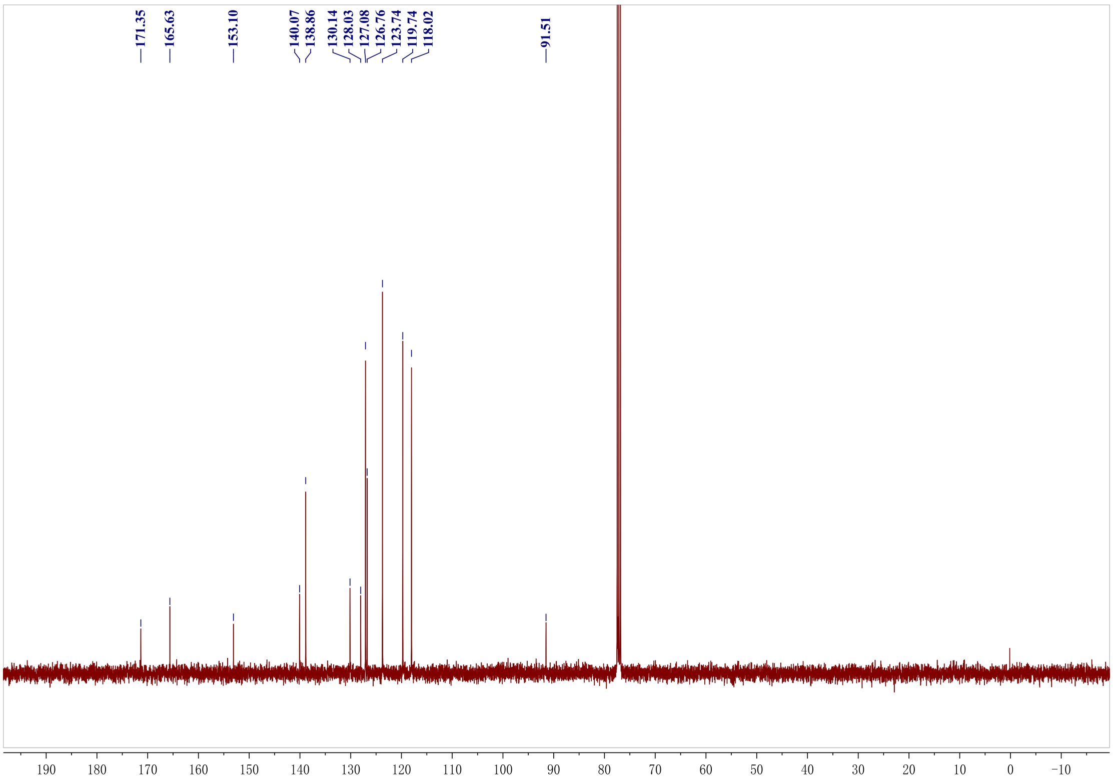

Supplementary Fig. 3 | The ^{13}C NMR spectrum of ITC molecule in $CDCl_3$.

III. Singlet oxygen generation in solution following X-ray irradiation.

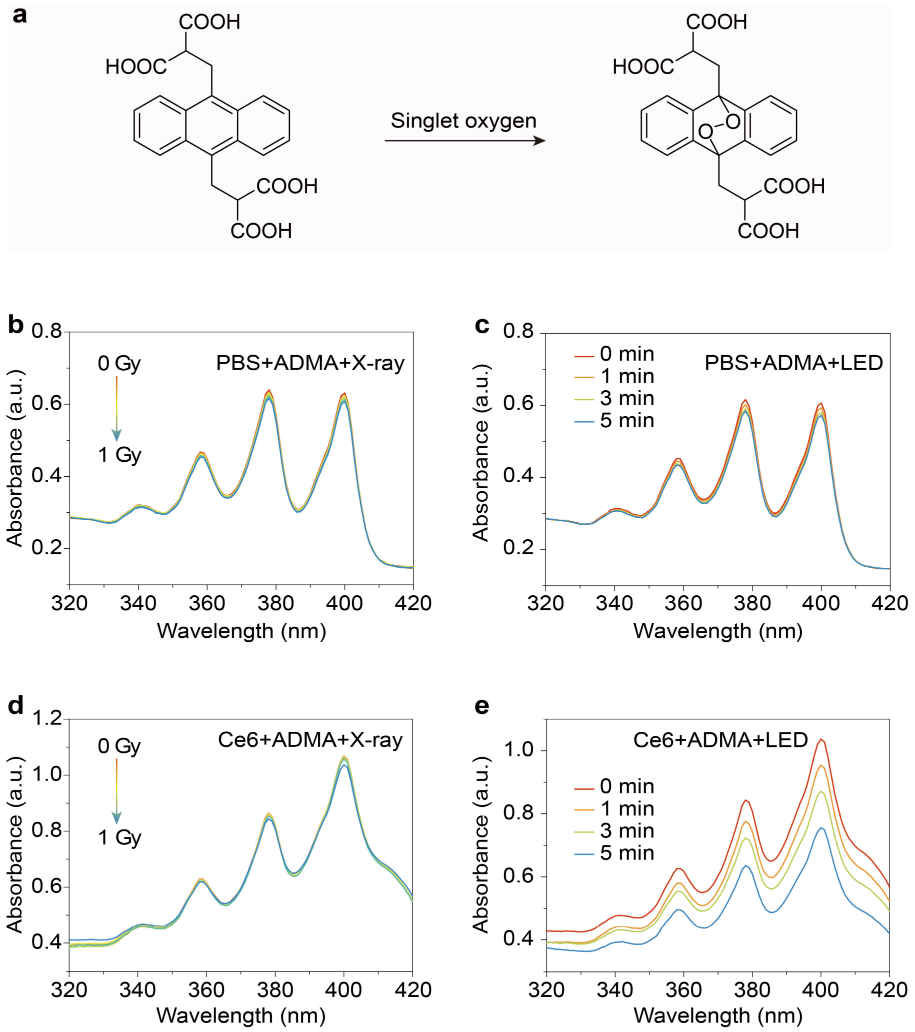

Supplementary Fig. 4 | $^1O_2$ generation ability of Ce6 in solution. The chemical structure of ADMA and its reaction with $^1O_2$ at room temperature (**a**). The related experiments of PBS (**b, c**) and Ce6 (**d, e**) solution in the presence of ADMA under X-ray irradiation (2.8 mGy/s, time interval: 72 seconds) or LED light (670 nm, 100 mW/cm^2, 300 s). Ce6 is the abbreviation of Chlorin E6 (CAS number: 19660-77-6).

IV. *In vitro* experiments.

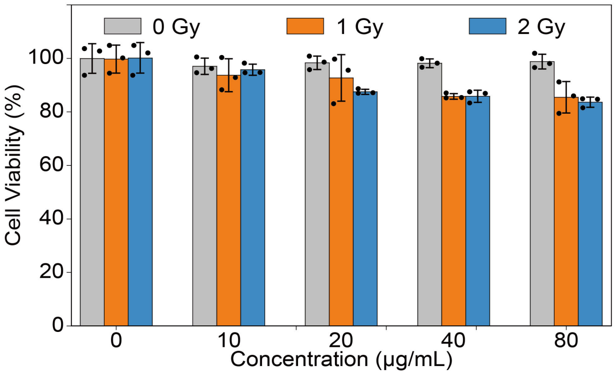

Supplementary Fig. 5 | Viabilities of 4T1 cells incubated with blank F127 micelles treated with or without X-ray radiation (1 or 2 Gy). Experiments were repeated for three times. The statistical data are expressed as mean values ± S.D. (n=3 independent experiments).

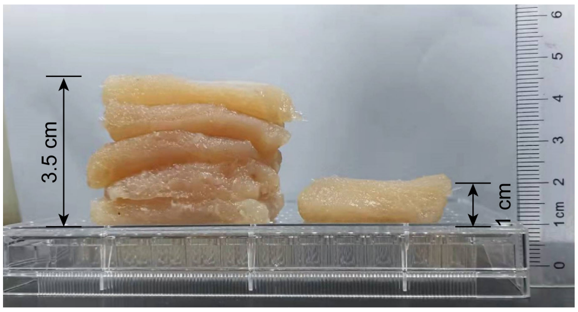

Supplementary Fig. 6 | Illustration of different depths of chicken breast. The X-ray or LED light was given from the upper of the chicken breast.

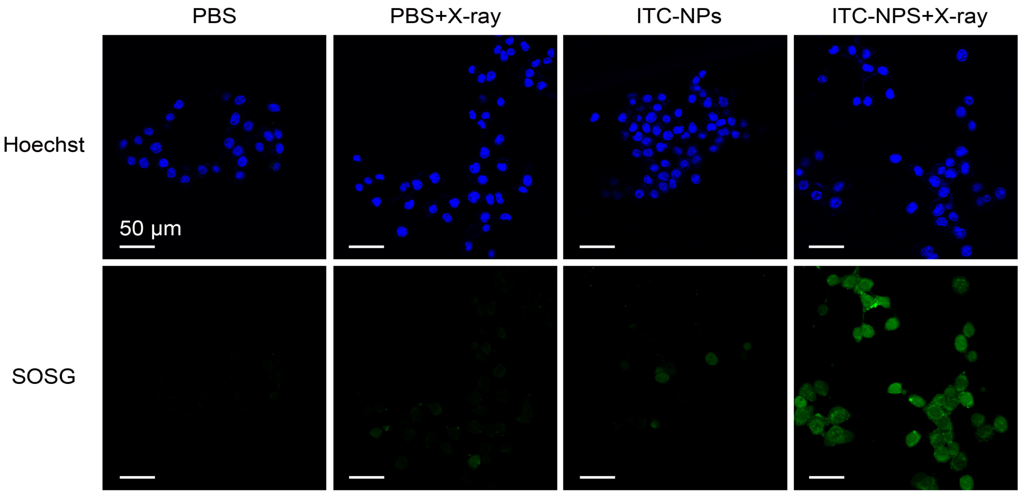

Supplementary Fig. 7 | CLSM images of 4T1 cells. The cells were stained by SOSG after incubating with PBS or scintillator nanoparticles for 24 hours with or without X-ray irradiation (2 Gy). The green fluorescence indicates the presence of $^1O_2$.

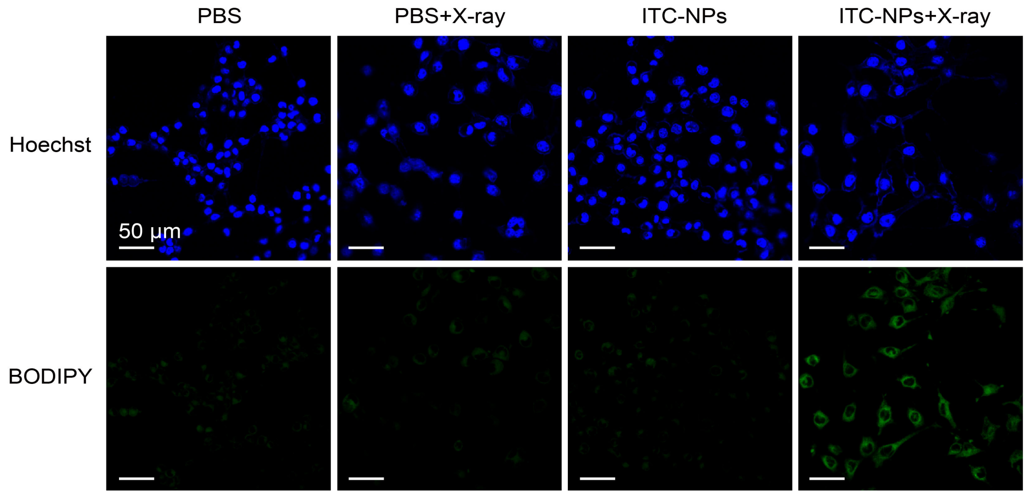

Supplementary Fig. 8 | CLSM images of lipoperoxides in 4T1 cells. The cells were incubated with PBS or scintillator nanoparticles for 24 hours with or without X-ray irradiation (2 Gy). The green fluorescence is the lipid ROS after the staining with BODIPY-C11.

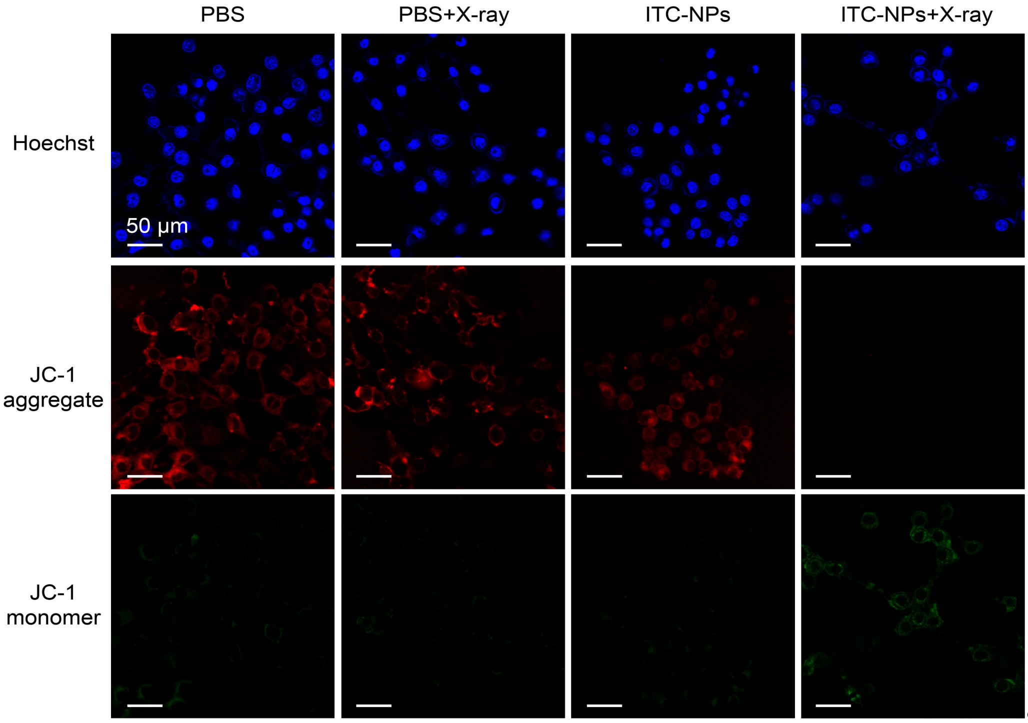

Supplementary Fig. 9 | CLSM images of the changes in the mitochondrial membrane potential of 4T1 cells. The cells were incubated with PBS or scintillator nanoparticles for 24 hours with or without X-ray irradiation (2 Gy). The red fluorescence indicates the membrane potential is positive and the green fluorescence indicated the membrane potential decreases.

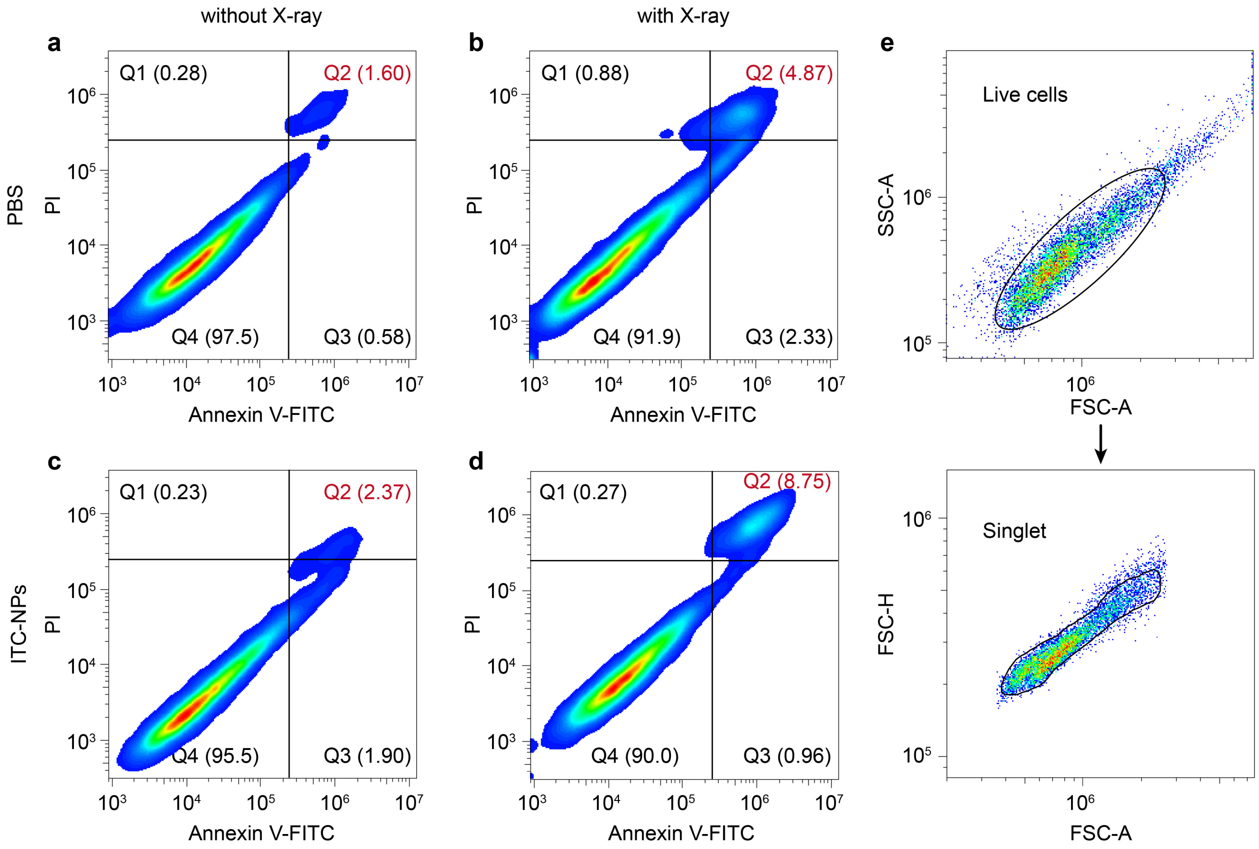

Supplementary Fig. 10 | Flow cytometric analysis image after various treatments. FITC Annexin V Apoptosis Detection Kit with PI was applied to identify the dead cells. For this kit, the Q2 area implies the percentage of dead cells. Among all the treatments, PBS+X-ray (**b**) and X-PDT (**d**) groups showed higher cytotoxicity on 4T1 tumor cells, with the latter one having advantage. The ITC-NPs+X-ray treatment was named as X-PDT group. (**e**) Gating strategies for the cell experiments in (**a-d**).

Gating strategies are referred to the method described in the BIO-RAD website (https://www.bio-rad-antibodies.com/flow-cytometry-gating-strategies.html). Gating was based on FSC/SCC together with fluorescent dyes and singlet populations. A forward-scatter (FSC) vs side-scatter (SSC) gate was used to gated on 4T1 cells to exclude debris. FSC-H vs FSC-A gate was used to gated on 4T1 singlet cells. The cell populations within the gate were analyzed based on the expression of markers.

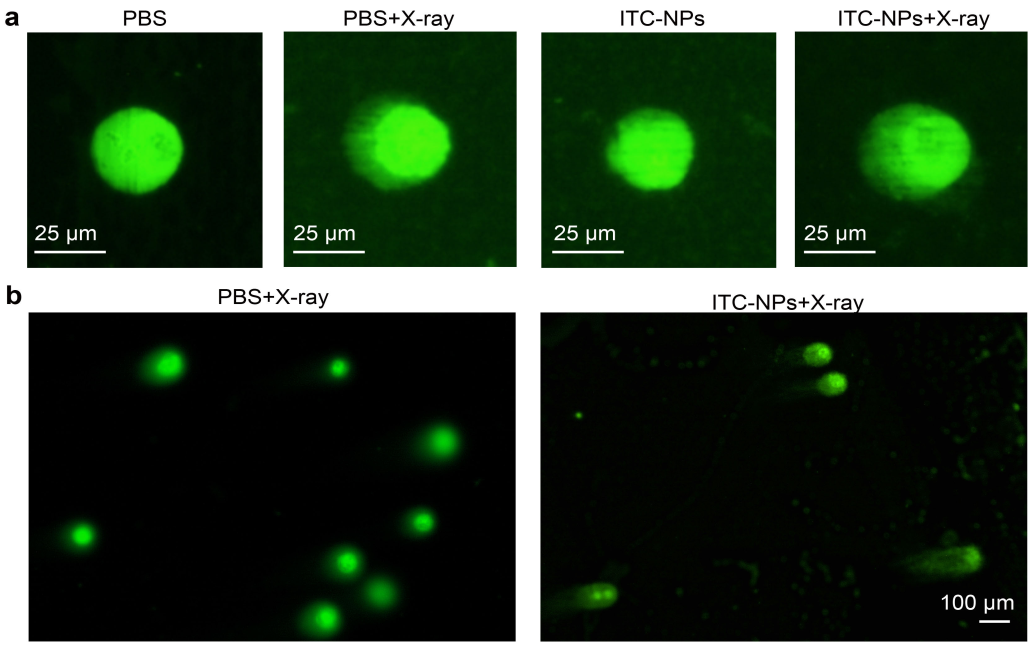

Supplementary Fig. 11 | DNA damage evaluation in 4T1 single cell (a) and 4T1 cells (b) by comet assays. The radiotherapeutic effect was evaluated through comet assay. As can be seen, single cell electrophoresis (i.e., comet assay) showed that a nucleoid with a spherical shape existed in blank control group and ITC-NPs group, indicating no DNA migration. In both the radiotherapy (PBS+X-ray) and X-PDT (ITC-NPs+X-ray) groups, strand breaks were found to form a comet-like appearance. Compared to radiotherapy (PBS+X-ray), X-PDT (ITC-NPs+X-ray) group induced a higher frequency of strand breaks, and formed loose and bondless nucleoid.

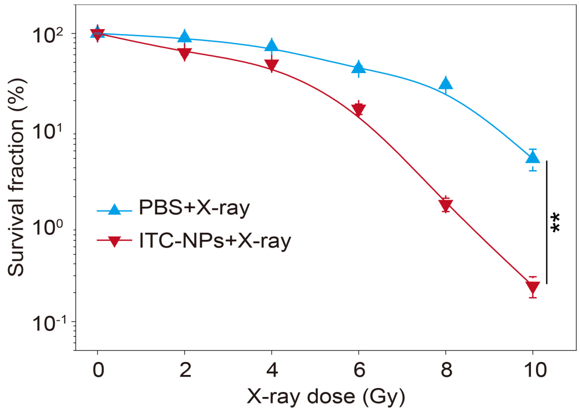

Supplementary Fig. 12 | Cell proliferation ability measured by clonogenic assay taken 14 days after radiotherapy (PBS+X-ray) or X-PDT (ITC-NPs+X-ray) treatments. The statistical data are expressed as mean values ± S.D. (n=3 independent experiments, $^{**}P = 0.0028$). Statistical significance was assessed via unpaired two-sided Student t-test.

V. Long-term toxicity evaluation.

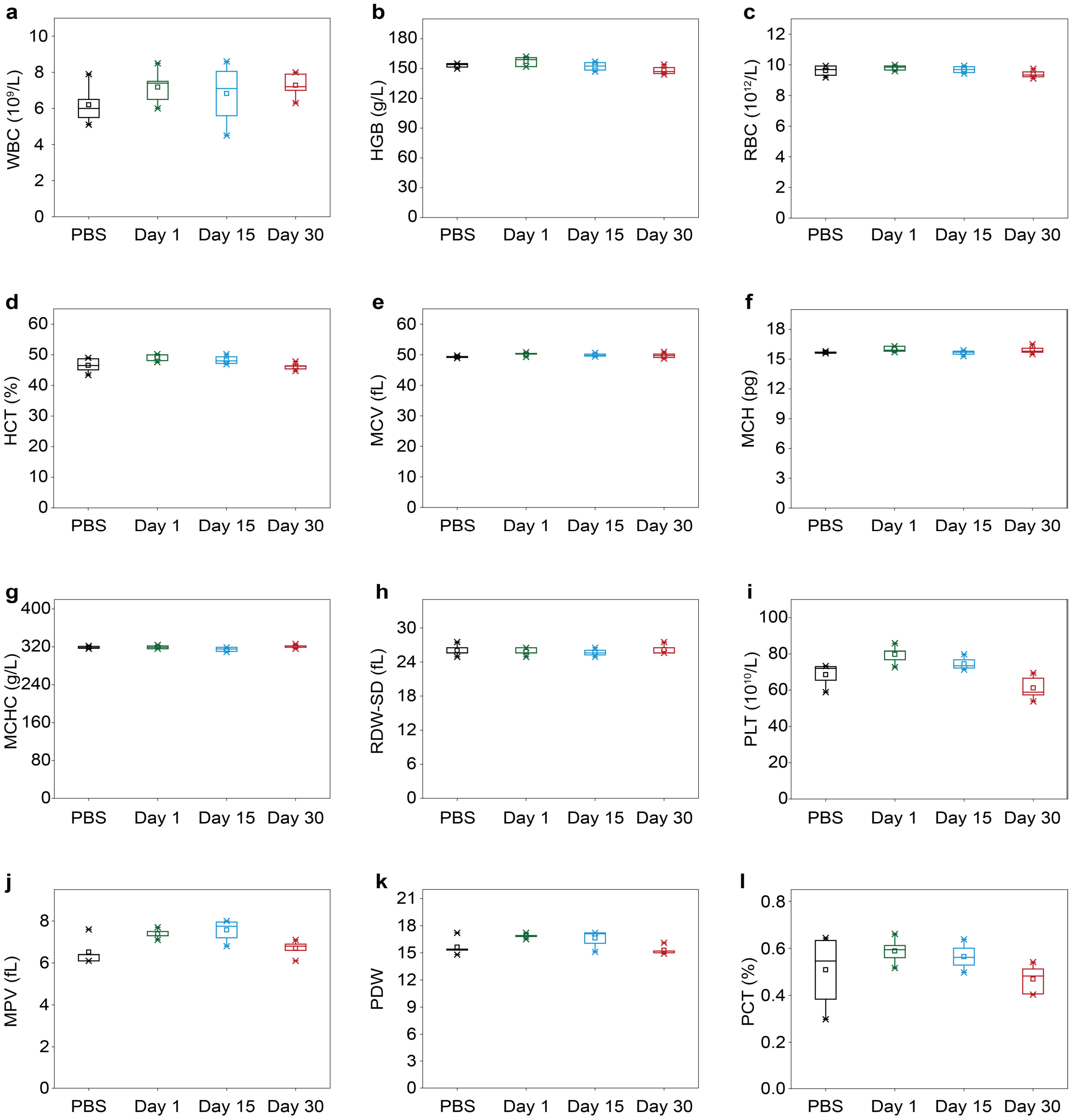

Supplementary Fig. 13 | Hematological analysis of mice treated with PBS or ITC-NPs. a, WBC, White blood cell. **b**, HGB, Haemoglobin. **c**, RBC, Red blood cell. **d**, HCT, Hematocrit. **e**, MCV, Mean corpuscular volume. **f**, MCH, Mean corpuscular hemoglobin. **g**, MCHC, Mean corpuscular hemoglobin concentration. **h**, RDW-SD, RBC distribution width. **i**, PLT, Platelets. **j**, MPV, Mean platelet volume. **k**, PDW, Platelet distribution width. **l**, PCT, Thrombocytocrit. Mice were intravenously treated daily for 3 days with PBS or nanoscintillator (50 mg kg^{-1}). Blood samples were collected for hematological analysis on day 1, day 15, and day 30 after treatment. Median and interquartile ranges are presented for the box plot (n=5 independent experiments).

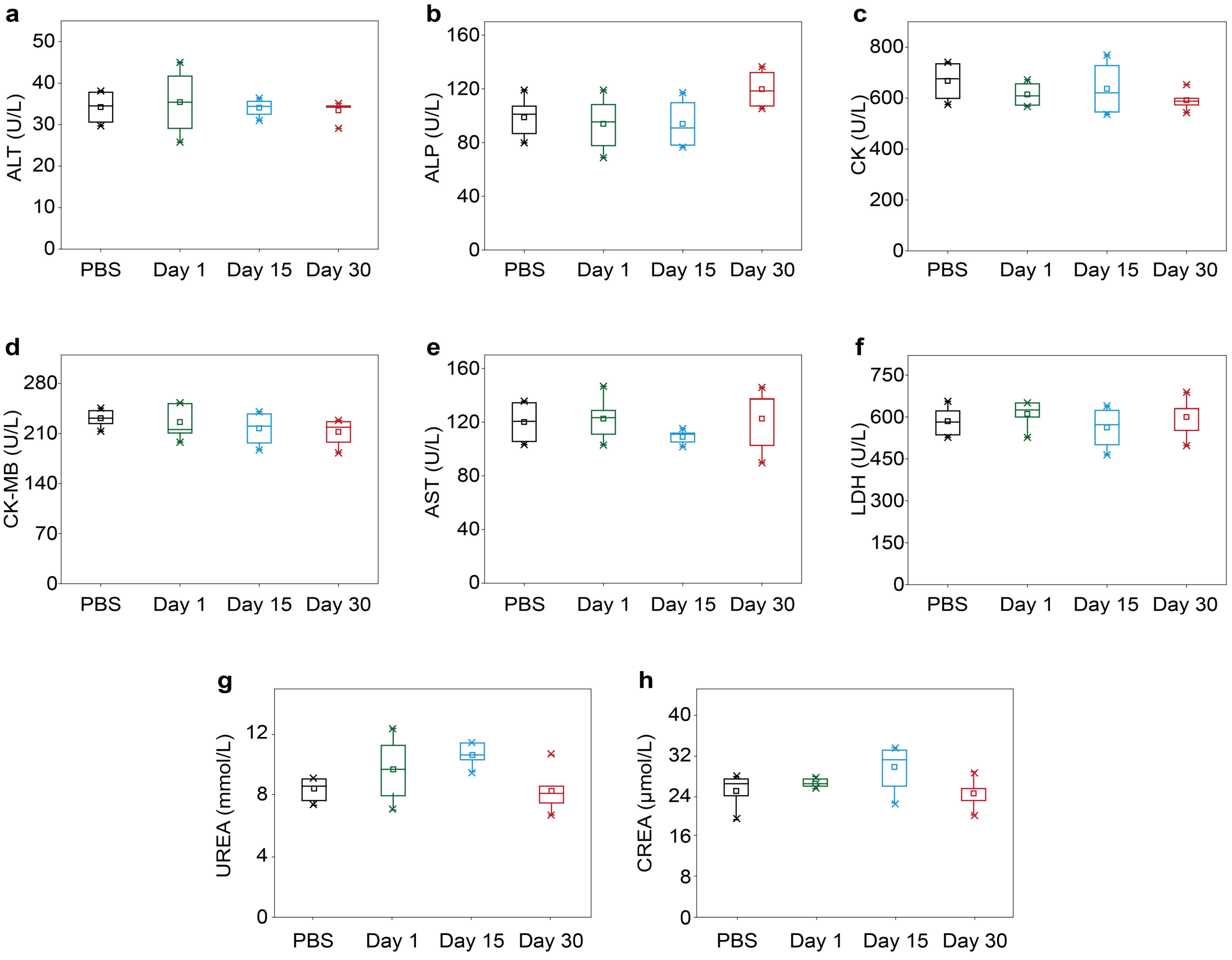

Supplementary Fig. 14 | Serum chemistry of mice treated with PBS or ITC-NPs. a, ALT, alanine transaminase. **b**, ALP, alkaline Phosphatase. **c**, CK, creatine kinase. **d**, CK-MB, creatine kinase mb isoenzyme. **e**, AST, aspartate transaminase. **f**, LDH, lactate dehydrogenase. **g**, UREA, blood urea nitrogen. **h**, CREA, creatinine. Mice were intravenously treated daily for 3 days with PBS or nanoscintillator (50 mg kg^{-1}). Blood samples were collected for serum chemistry analysis on day 1, day 15, and day 30 after treatment. Median and interquartile ranges are presented for the box plot (n=5 independent experiments).

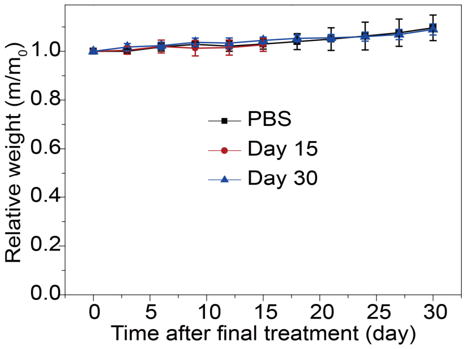

Supplementary Fig. 15 | The mice body weights of mice treated with different strategies. The statistical data are expressed as mean values ± S.D. (n=5 independent animals).

VI. *In vivo* fluorescent imaging and clearance behaviours.

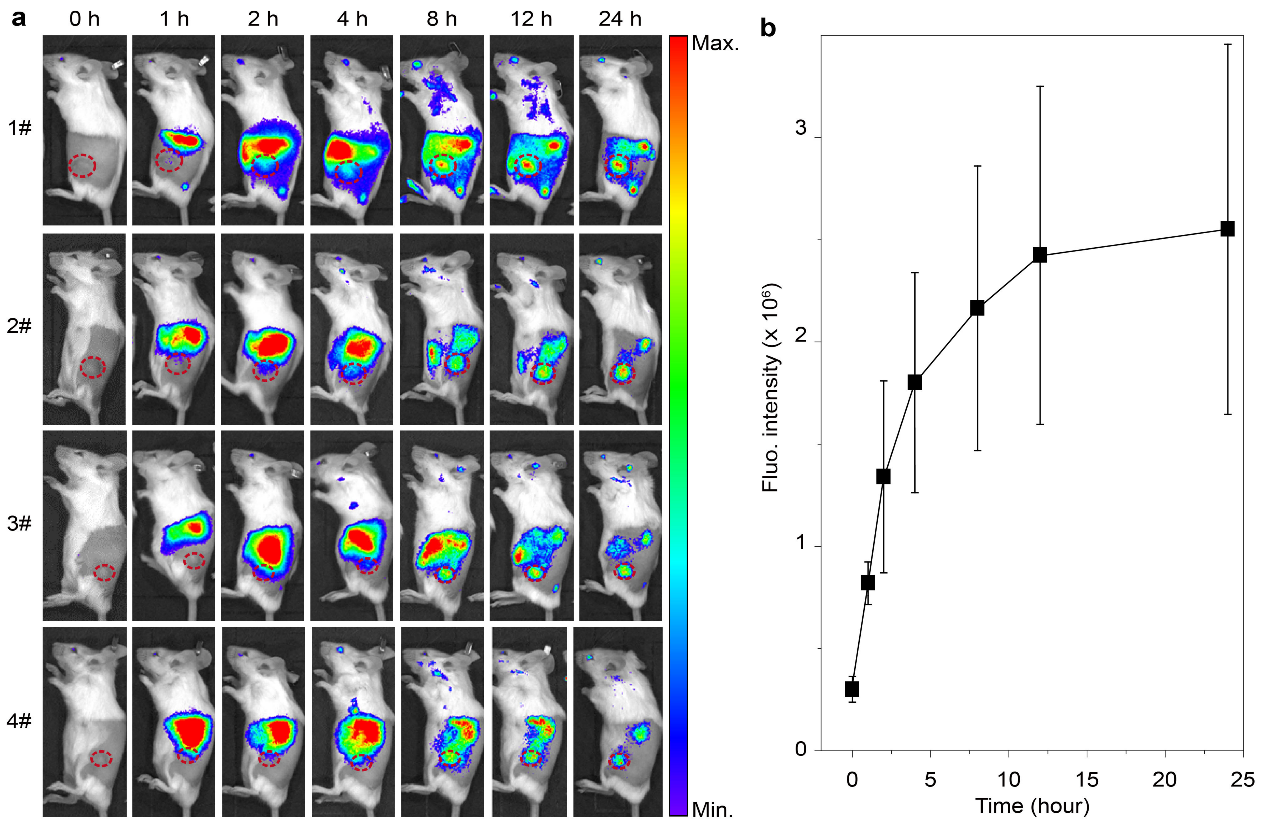

Supplementary Fig. 16 | Accumulation of nanoscintillators in 4T1 tumours. a, The *in vivo* fluorescent images of 4T1 tumour-bearing mice. The red dotted circles indicate the subcutaneous tumour regions (1#, 2#, 3#, 4#: four parallel animal). **b**, Corresponding fluorescence intensity in tumour regions at 0, 1, 2, 4, 8, 12, and 24 h post-injection of nanoscintillators labeled with Cy5.5-COOH. The statistical data are expressed as mean values ± S.D. (n=4 independent animals).

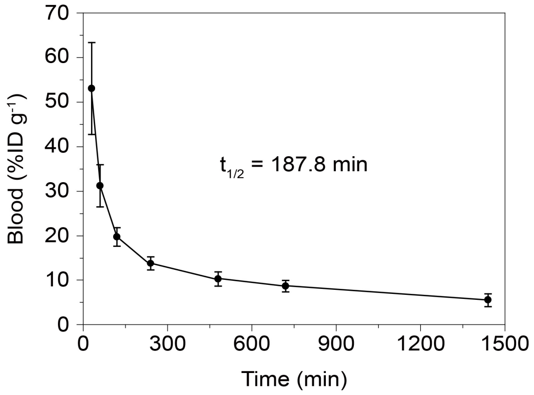

Supplementary Fig. 17 | Blood-circulation (%ID g^{-1}) of nanoscintillators labeled with Cy5.5-COOH as a function of time. The statistical data are expressed as mean values ± S.D. (n=3 independent animals).

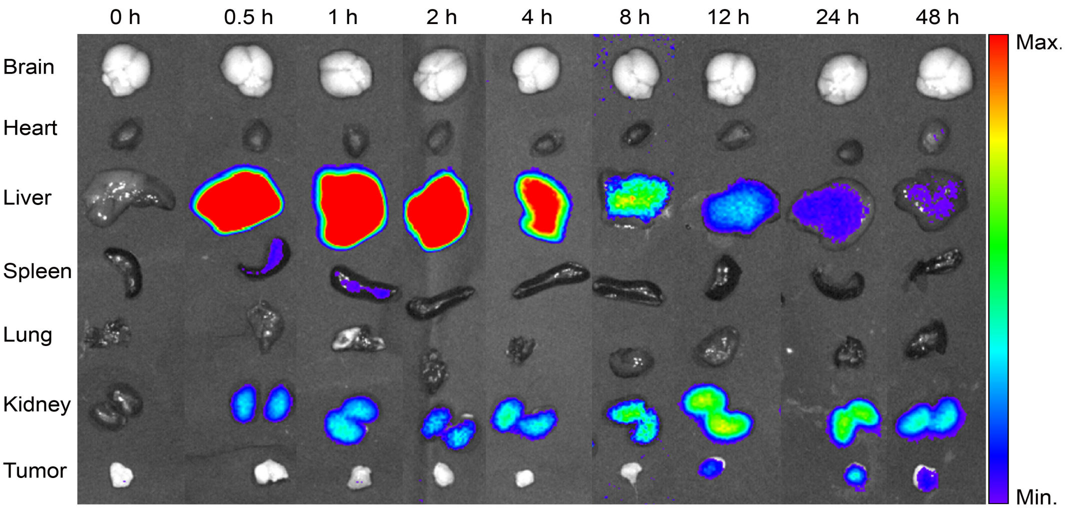

Supplementary Fig. 18 | The *ex vivo* fluorescence imaging of 4T1 tumour-bearing mice after intravenous injection of nanoscintillators labeled with Cy5.5-COOH.

VII. *In vivo* experiments *via* intratumoral injection.

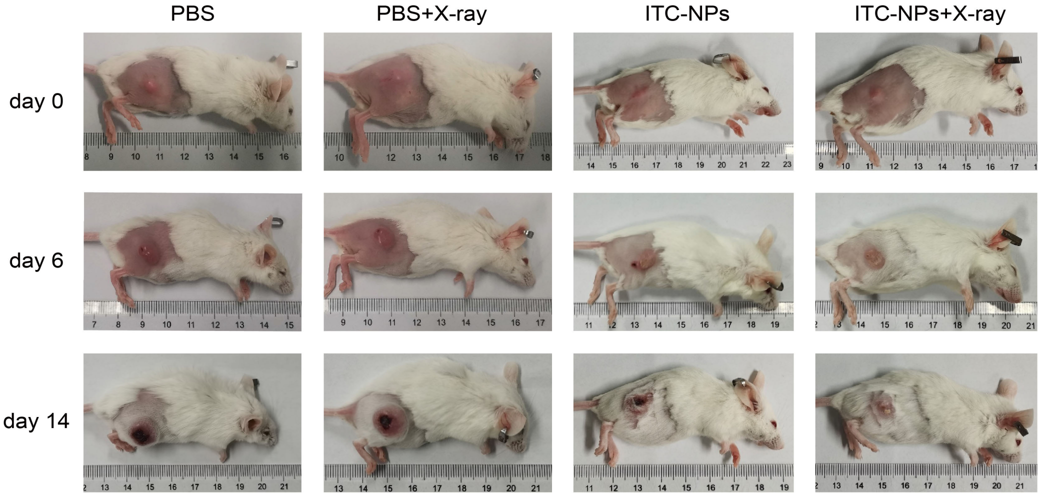

Supplementary Fig. 19 | Representative mice photographs at days 0, 6, and 14, after different intratumoral treatment groups.

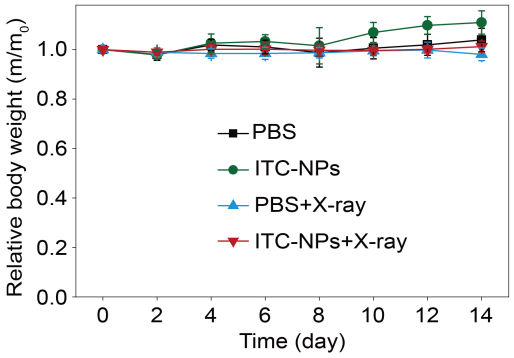

Supplementary Fig. 20 | The mice body weights of mice after various intratumoral treatments. The statistical data

are expressed as mean values ± S.D. (n=6 independent animals).

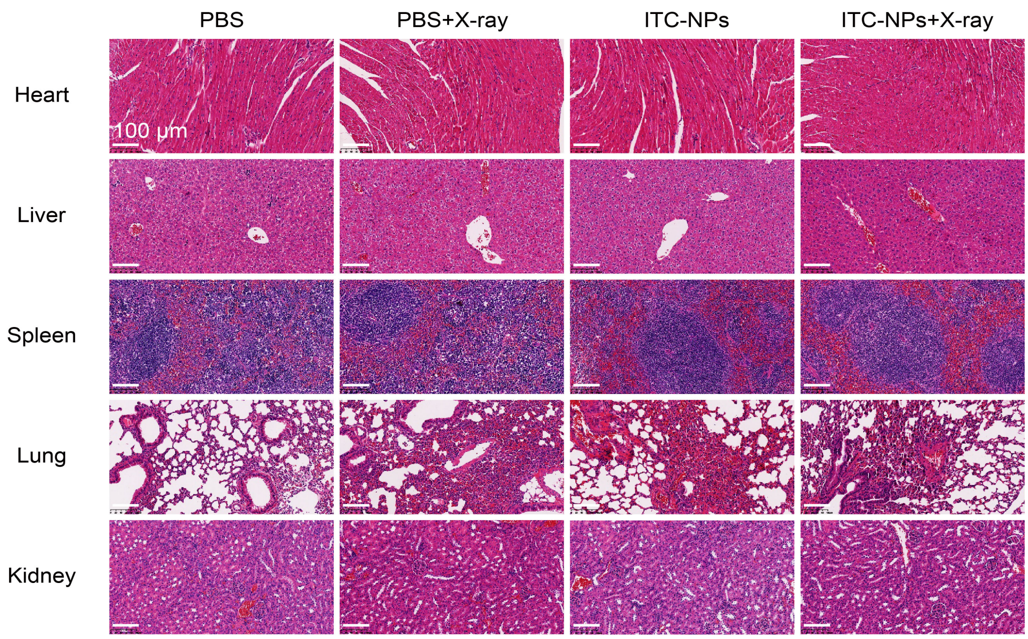

Supplementary Fig. 21 | H&E-stained images of main normal tissues. The tissues include heart, liver, spleen, lung, and kidney slices (scale bar, 100 μm), after various intratumoral treatments.

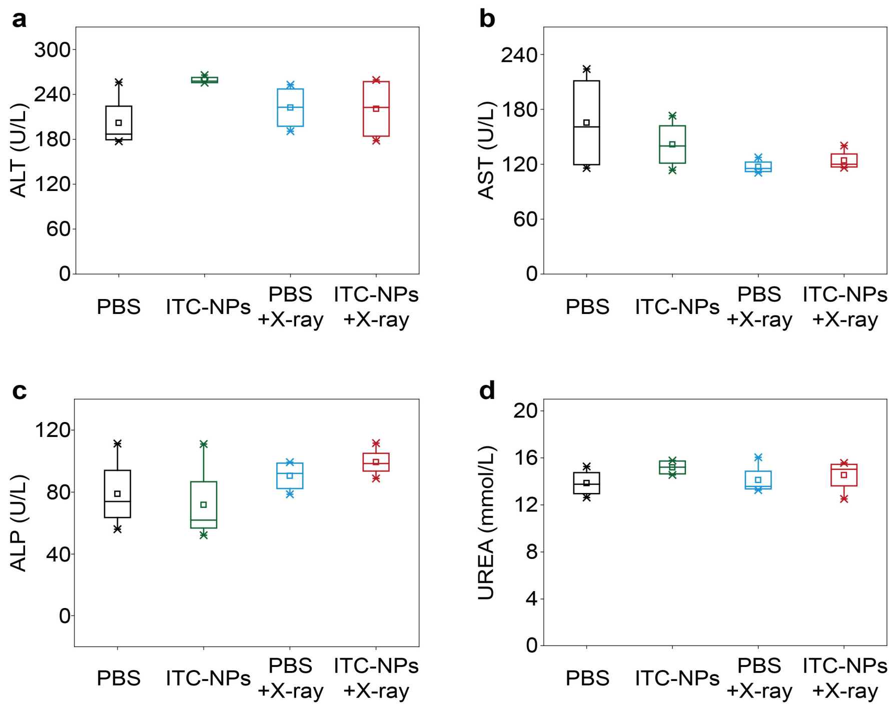

Supplementary Fig. 22 | Serum chemistry of mice with different treatments with PBS, ITC-NPs, PBS+X-ray, or ITC-NPs+X-ray. a, ALT, alanine transaminase. **b**, AST, aspartate transaminase. **c**, ALP, alkaline phosphatase. **d**, UREA, blood urea nitrogen. Mice were intratumorally treated with PBS or scintillator nanoparticles (0.4 mg/mL, 200 μL). Blood

samples were collected for serum chemistry analysis on day 14 after treatment. Median and interquartile ranges are presented for the box plot (n=4 independent animals).

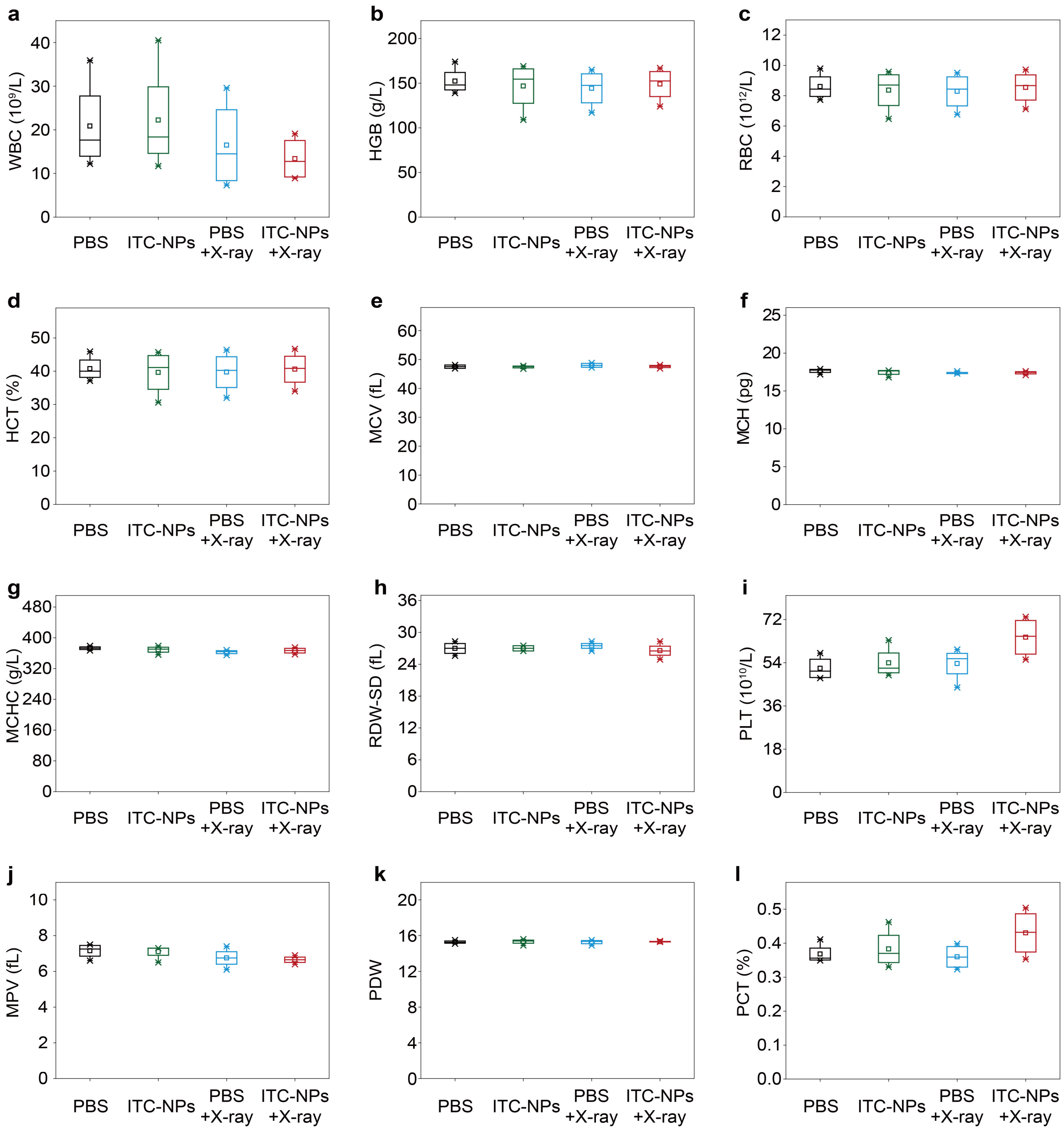

Supplementary Fig. 23 | Hematological analysis of mice treated with PBS, ITC-NPs, PBS+X-ray, or ITC-NPs+X-ray. **a**, White blood cell (WBC). **b**, Haemoglobin (HGB). **c**, Red blood cell (RBC). **d**, Hematocrit (HCT). **e**, Mean corpuscular volume (MCV). **f**, Mean corpuscular hemoglobin (MCH). **g**, Mean corpuscular hemoglobin concentration (MCHC). **h**,

RBC distribution width (RDW-SD). **i**, Platelets (PLT). **j**, Mean platelet volume (MPV). **k**, Platelet distribution width (PDW). **l**, Thrombocytocrit (PCT). Mice were intratumorally treated with PBS or scintillator nanoparticles (0.4 mg/mL, 200 μL). Blood samples were collected for hematological analysis on day 14 after treatment. Median and interquartile ranges are presented for the box plot (n=4 independent animals).

VIII. *In vivo* experiments *via* intravenous injection.

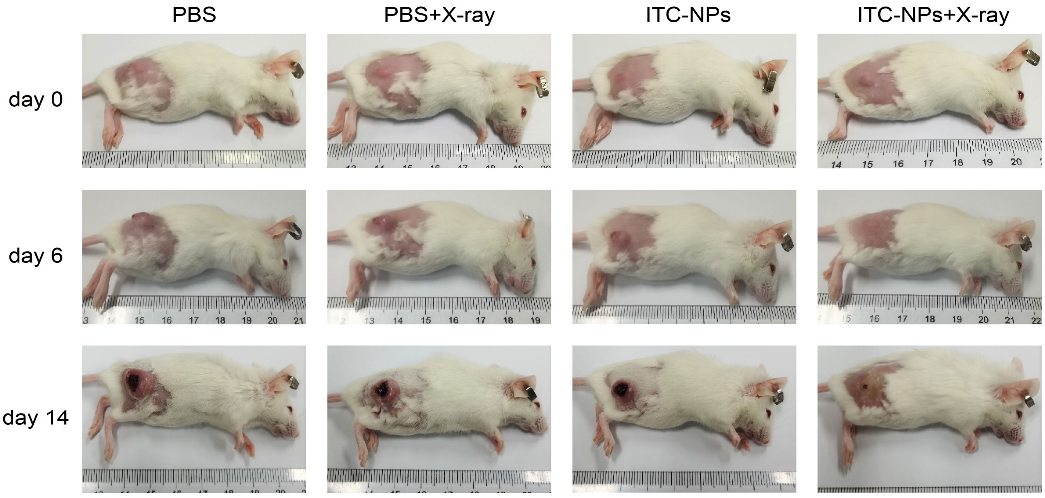

Supplementary Fig. 24 | Representative mice photographs at days 0, 6, and 14, after different intravenous treatment groups.

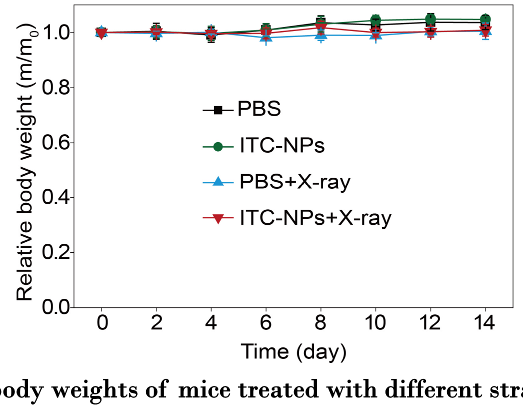

Supplementary Fig. 25 | The mice body weights of mice treated with different strategies. The statistical data are expressed as mean values ± S.D. (n=5 independent animals).

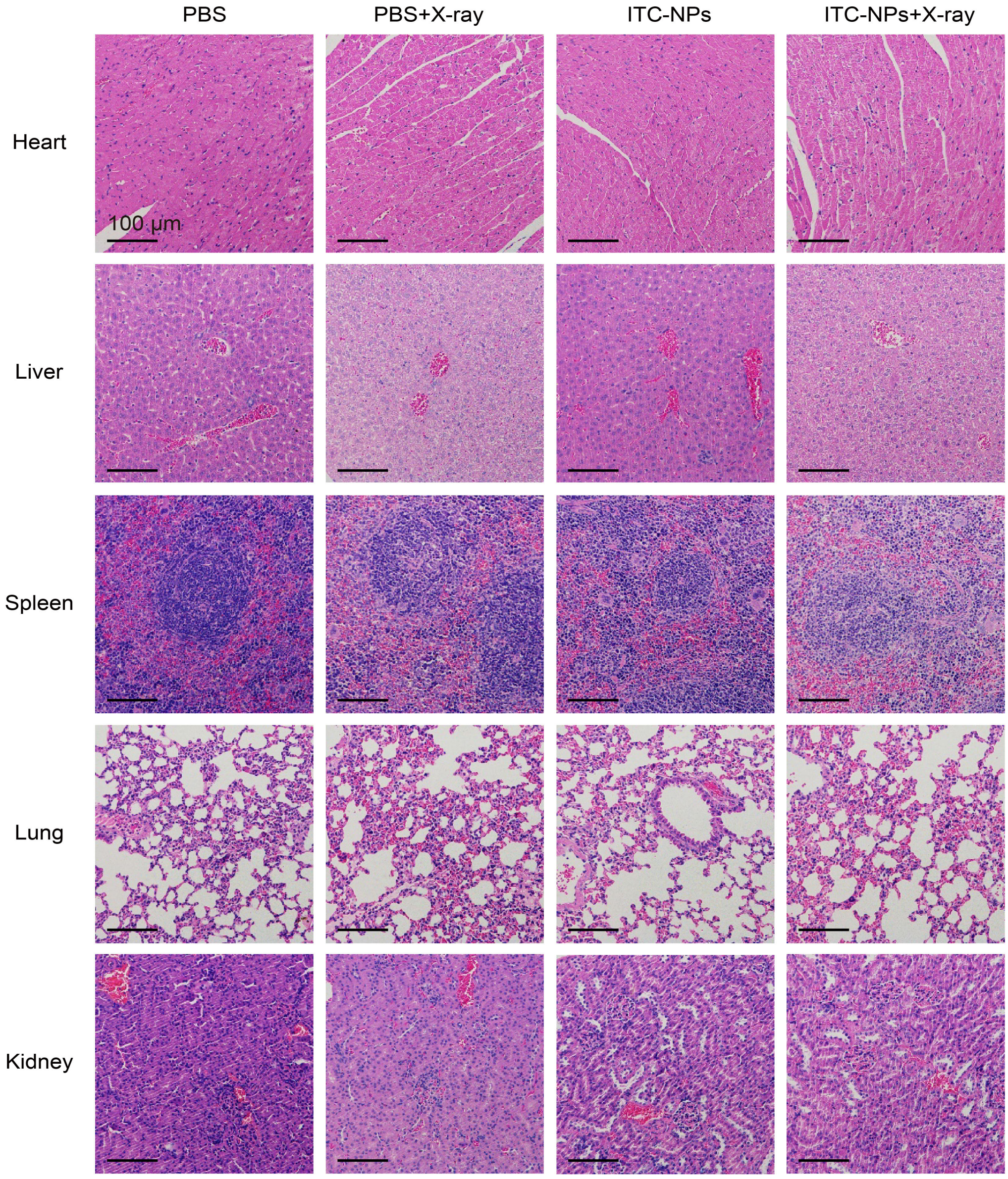

Supplementary Fig. 26 | H&E-stained images of main normal tissues. The tissues include heart, liver, spleen, lung, and kidney slices (scale bar, 100 μm), after various intravenous treatments.

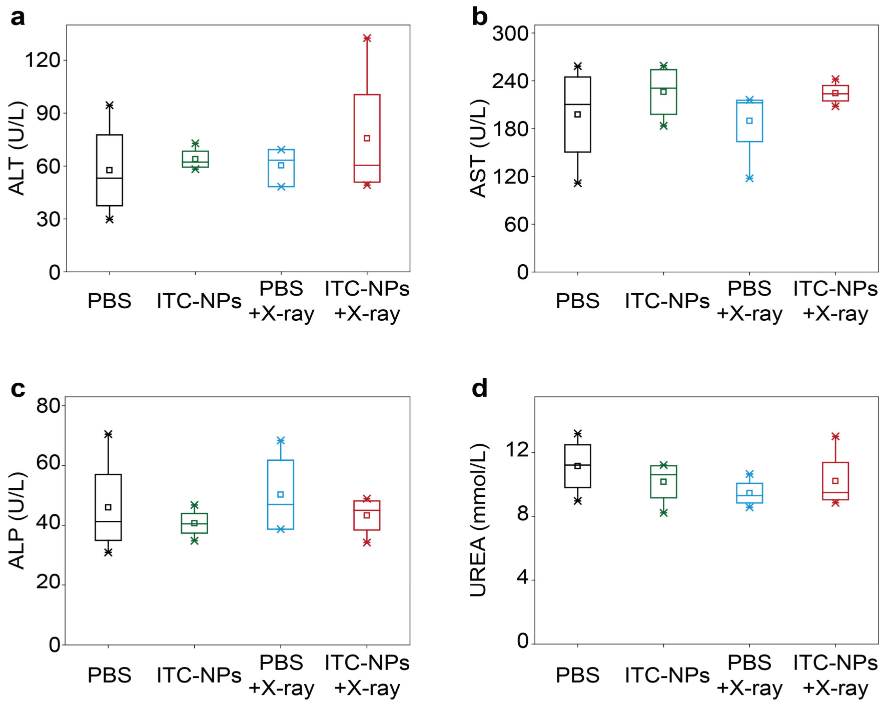

Supplementary Fig. 27 | Serum chemistry of mice with different treatments with PBS, ITC-NPs, PBS+X-ray, or ITC-NPs+X-ray. **a**, ALT, alanine transaminase. **b**, AST, aspartate transaminase. **c**, ALP, alkaline phosphatase. **d**, UREA, blood urea nitrogen. Mice were intravenously treated with PBS or scintillator nanoparticles (0.4 mg/mL, 200 μL). Blood samples were collected for serum chemistry analysis on day 14 after treatment. Median and interquartile ranges are presented for the box plot (n=4 independent animals).

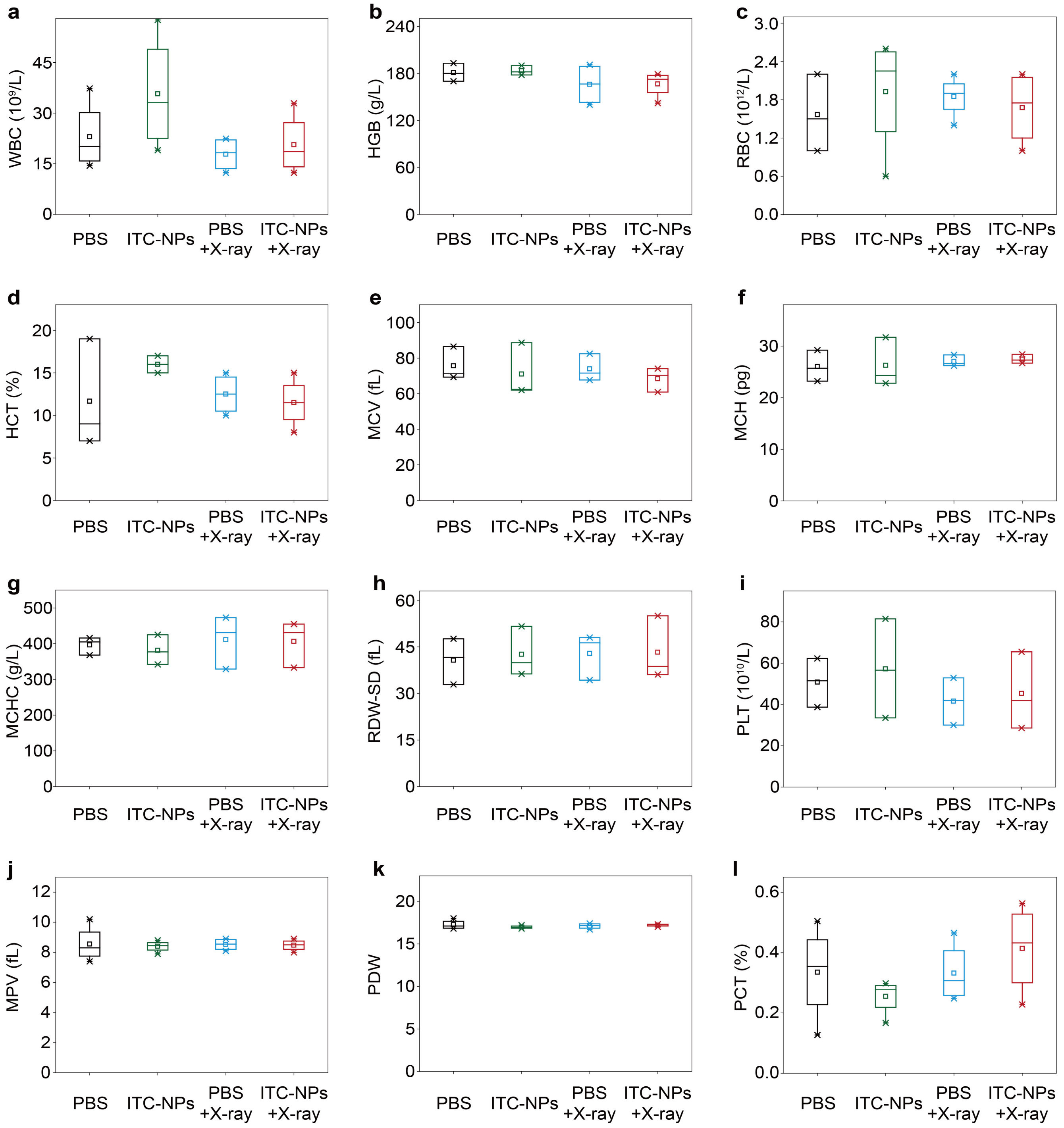

Supplementary Fig. 28 | Hematological analysis of mice treated with PBS, ITC-NPs, PBS+X-ray, or ITC-NPs+X-ray. **a**, White blood cell (WBC). **b**, Haemoglobin (HGB). **c**, Red blood cell (RBC). **d**, Hematocrit (HCT). **e**, Mean corpuscular volume (MCV). **f**, Mean corpuscular hemoglobin (MCH). **g**, Mean corpuscular hemoglobin concentration (MCHC). **h**, RBC distribution width (RDW-SD). **i**, Platelets (PLT). **j**, Mean platelet volume (MPV). **k**, Platelet distribution width (PDW). **l**, Thrombocytocrit (PCT). Mice were intravenously treated with PBS or scintillator nanoparticles (0.4 mg/mL, 200 μL). Blood samples were collected for hematological analysis on day 14 after treatment. Median and interquartile

ranges are presented for the box plot (n=4 independent animals).

IX. References

1. Liu, Y., Chen, W., Wang, S. & Joly, A. Investigation of water-soluble x-ray luminescence nanoparticles for photodynamic activation. *Appl. Phys. Lett.* **92**, 043901 (2008).
2. Takahashi, J. & Misawa, M. Analysis of potential radiosensitizing materials for x-ray-induced photodynamic therapy. *NanoBiotechnol* **3**, 116-126 (2008).
3. Abliz, E., Collins, J., Bell, H. & Tata, D. Novel applications of diagnostic X-rays in activating a clinical photodynamic drug: photofrin ii through x-ray induced visible luminescence from "rare-earth" formulated particles. *J. X-ray. Sci. Technol.* **19**, 521-530 (2011).
4. Scaffidi, J., Gregas, M., Lauly, B., Zhang, Y. & Vo-Dinh, T. Activity of psoralen-functionalized nanoscintillators against cancer cells upon X-ray excitation. *ACS Nano* **5**, 4679-4687 (2011).
5. Bulin, A. et al. X-ray-induced singlet oxygen activation with nanoscintillator-coupled porphyrins. *J. Phys. Chem. C* **117**, 21583-21589 (2013).
6. Ma, L. et al. X-ray excited ZnS:Cu,Co afterglow nanoparticles for photodynamic activation. *Appl. Phys. Lett.* **105**, 013702 (2014).
7. Zhang, C. et al. Marriage of scintillator and semiconductor for synchronous radiotherapy and deep photodynamic therapy with diminished oxygen dependence. *Angew. Chem. Int. Ed.* **54**, 1770-1774 (2015).
8. Tang, Y., Hu, J., Elmenoufy, A. & Yang, X. Highly efficient FRET system capable of deep photodynamic therapy established on X-ray excited mesoporous LaF3:Tb scintillating nanoparticles. *ACS Appl. Mater. Interfaces.* **7**, 12261-12269 (2015).
9. Kirakci, K. et al. X-ray inducible luminescence and singlet oxygen sensitization by an octahedral molybdenum cluster compound: a new class of nanoscintillators. *Inorg. Chem.* **55**, 803-809 (2016).
10. Chen, H. et al. Nanoscintillator-mediated X-ray inducible photodynamic therapy for in vivo cancer treatment. *Nano Lett.* **15**, 2249-2256 (2015).
11. Kaščáková, S. et al. X-ray-induced radiophotodynamic therapy (RPDT) using lanthanide micelles: beyond depth limitations. *Nano Res.* **8**, 2373-2379 (2015).
12. Elmenoufy AH, Tang Y, Hu J, Xu H, Yang X. A novel deep photodynamic therapy modality combined with ct imaging established via X-ray stimulated silica-modified lanthanide scintillating nanoparticles. *Chem. Commun.* **51**, 12247-12250 (2015).
13. Wang, G. et al. X-ray induced photodynamic therapy: a combination of radiotherapy and photodynamic therapy. *Theranostics* **6**, 2295-2305 (2016).
14. Lan, G. et al. Nanoscale metal-organic layers for deeply penetrating x-ray-induced photodynamic therapy. *Angew. Chem. Int. Ed.* **56**, 12102-12106 (2017).
15. Chen, H. et al. LiGa5O8:Cr-based theranostic nanoparticles for imaging-guided x-ray induced photodynamic therapy of deep-seated tumors. *Mater. Horiz.* **4**, 1092-1101 (2017).
16. Ni, K. et al. Nanoscale Metal-organic frameworks for mitochondria-targeted radiotherapy-radiodynamic therapy. *Nat. Commun.* **9**, 4321 (2018).
17. Lan, G. et al. Nanoscale metal-organic framework overcomes hypoxia for photodynamic therapy primed cancer immunotherapy. *J. Am. Chem. Soc.* **140**, 5670-5673 (2018).
18. Lu, K. et al. Low-Dose x-ray radiotherapy–radiodynamic therapy via nanoscale metal–organic frameworks enhances

checkpoint blockade immunotherapy. *Nat. Biomed. Eng.* **2**, 600-610 (2018).
19. Wang, H. et al. Scintillator-based nanohybrids with sacrificial electron prodrug for enhanced X-ray-induced photodynamic therapy. *Nano Lett.* **18**, 5768-5774 (2018).
20. Song, L. et al. Low-Dose X-Ray Activation of W(VI)-doped persistent luminescence nanoparticles for deep-tissue photodynamic therapy. *Adv. Funct. Mater.* **28**, 1707496 (2018).
21. Shrestha, S. et al. X-ray induced photodynamic therapy with copper-cysteamine nanoparticles in mice tumors. Proc. Natl. Acad. Sci. USA **116**, 16823-16828 (2019).
22. Yu, X. et al. CT/MRI-guided synergistic radiotherapy and X-ray inducible photodynamic therapy using Tb-doped Gd-W-nanoscintillators. *Angew. Chem. Int. Ed.* **58**, 2017-2022 (2019).
23. Sun, W. et al. Monodisperse and uniform mesoporous silicate nanosensitizers achieve low-dose X-ray-induced Deep-penetrating photodynamic therapy. *Adv. Mater.* **31**, 1808024 (2019).
24. Sun, W. et al. Aggregation-induced emission gold clustoluminogens for enhanced low-dose X-ray-induced photodynamic therapy. *Angew. Chem. Int. Ed.* **59**, 9914-9921 (2020).
25. Deng, W. et al. Application of mitochondrially targeted nanoconstructs to neoadjuvant X-ray-induced photodynamic therapy for rectal cancer. *ACS Cent. Sci.* **6**, 715-726 (2020).
26. Gu, X., Shen, C., Li, H., Goldys, E. & Deng, W. X-ray induced photodynamic therapy (PDT) with a mitochondria-targeted liposome delivery system. *J. Nanobiotechnol.* **18**, 87 (2020).
27. Sah, B. et al. Effects of Nanoparticle size and radiation energy on copper-cysteamine nanoparticles for X-ray induced photodynamic therapy. *Nanomaterials* **10**, 1087 (2020).
28. Sang, W. et al. Oxygen-enriched metal-phenolic X-ray nanoprocessor for cancer radio-radiodynamic therapy in combination with checkpoint blockade immunotherapy. *Adv. Sci.* **8**, 2003338 (2021).
29. Liu, J. et al. Bioorthogonal Coordination Polymer Nanoparticles with Aggregation-Induced Emission for Deep Tumor-Penetrating Radio- and Radiodynamic Therapy. *Adv. Mater.* **33**, 2007888 (2021).
30. Rossi, F. et al. Porphyrin conjugated SiC/SiOx nanowires for X-ray-excited photodynamic therapy. *Sci. Rep.* **5**, 7606 (2015).
31. Wang, X. et al. Organic phosphors with bright triplet excitons for efficient X-ray-excited luminescence. *Nat. Photon.* **15**, 187-192 (2021).
