# Supplementary material for: Organic phosphorescent nanoscintillator for low-dose X-ray-induced photodynamic therapy
Source: Nat Commun. 2022 Aug 30;13:5091. doi: 10.1038/s41467-022-32054-0 (PMC9428140; doi:10.1038/s41467-022-32054-0)
Supplement: Supplementary file 2 — Reporting Summary [file 41467_2022_32054_MOESM2_ESM.pdf]

nature portfolio

Corresponding author(s): Zhongfu An, Hongmin Chen, Wei Huang

Last updated by author(s): Jun 17, 2022

Reporting Summary

Nature Portfolio wishes to improve the reproducibility of the work that we publish. This form provides structure for consistency and transparency in reporting. For further information on Nature Portfolio policies, see our Editorial Policies and the Editorial Policy Checklist.

Statistics

For all statistical analyses, confirm that the following items are present in the figure legend, table legend, main text, or Methods section.

n/a	Confirmed	
☐	☒	The exact sample size (*n*) for each experimental group/condition, given as a discrete number and unit of measurement
☐	☒	A statement on whether measurements were taken from distinct samples or whether the same sample was measured repeatedly
☐	☒	The statistical test(s) used AND whether they are one- or two-sided<br>*Only common tests should be described solely by name; describe more complex techniques in the Methods section.*
☒	☐	A description of all covariates tested
☒	☐	A description of any assumptions or corrections, such as tests of normality and adjustment for multiple comparisons
☐	☒	A full description of the statistical parameters including central tendency (e.g. means) or other basic estimates (e.g. regression coefficient) AND variation (e.g. standard deviation) or associated estimates of uncertainty (e.g. confidence intervals)
☐	☒	For null hypothesis testing, the test statistic (e.g. *F*, *t*, *r*) with confidence intervals, effect sizes, degrees of freedom and *P* value noted<br>*Give P values as exact values whenever suitable.*
☒	☐	For Bayesian analysis, information on the choice of priors and Markov chain Monte Carlo settings
☒	☐	For hierarchical and complex designs, identification of the appropriate level for tests and full reporting of outcomes
☒	☐	Estimates of effect sizes (e.g. Cohen's *d*, Pearson's *r*), indicating how they were calculated

Our web collection on statistics for biologists contains articles on many of the points above.

Software and code

Policy information about availability of computer code

Data collection	Fluoracle® software for all steady state and time-resolved luminescence measurements.<br>NanoPlus-3 Particle Analyzer (OTSUKA) for zeta potential measurements.<br>Cary60 PC spectrophotometer for collecting the UV-vis absorption spectra.<br>Laser scanning confocal microscope (Olympus FV1200, Japan) for imaging fluorescence images of cells.<br>CytExpert software 2.0 for Flow-Cytometry method.
Data analysis	General data analysis: Origin 8.1, and software Image J2x V2.1.4.7. Quantitative results were presented as mean ± standard deviation. GraphPad Prism 7.00 was used to conduct statistical analysis. Student's T Test: The P value was analyzed by SPSS (Statistical Package for the Social Sciences, SPSS statistics 22.0). Fluoracle® software was used to analyse photoluminescence lifetimes.

For manuscripts utilizing custom algorithms or software that are central to the research but not yet described in published literature, software must be made available to editors and reviewers. We strongly encourage code deposition in a community repository (e.g. GitHub). See the Nature Portfolio guidelines for submitting code & software for further information.

Data

Policy information about availability of data

All manuscripts must include a data availability statement. This statement should provide the following information, where applicable:

- Accession codes, unique identifiers, or web links for publicly available datasets
- A description of any restrictions on data availability
- For clinical datasets or third party data, please ensure that the statement adheres to our policy

The authors declare that all data supporting the findings of this study are provided in the Supplementary Information/Source Data file. Source data are provided

with this paper.

Field-specific reporting

Please select the one below that is the best fit for your research. If you are not sure, read the appropriate sections before making your selection.

☒ Life sciences ☐ Behavioural & social sciences ☐ Ecological, evolutionary & environmental sciences

For a reference copy of the document with all sections, see nature.com/documents/nr-reporting-summary-flat.pdf

Life sciences study design

All studies must disclose on these points even when the disclosure is negative.

Sample size

No statistical method was used to predetermine the sample size for each study. For property measurement experiments, samples were prepared and tested three times independently. In vitro studies, samples were repeated three times independently; For in vivo studies, each group contains at least 3 for evaluating the statistical significance.
For in vitro studies, the cancer cell density is about 10^5/mL, it is appropriate for each in vitro assay according to the reproduction and growth rate of the cancer cells.
For in vivo studies, to avoid obvious individual differences, the mice bearing subcutaneous tumors are divided randomly into four groups with at least three mice in each group.

Data exclusions

No data was excluded from the study.

Replication

For X-ray-induced photodynamic therapy in mice, four independent experiments in each group were performed, and all attempts at replication were successful. For property measurement experiments, samples were replicated and tested independently for 3 times with standard deviation displayed, and all attempts at replication were successful.

Randomization

The mice that used for evaluating X-ray-induced photodynamic therapy were randomized by simple random sampling strategy.
For experiments other than those involving mice, the samples were allocated randomly into experimental groups, such as the 4T1 cell sample and the ITC NPs sample etc.

Blinding

The investigators were not blinded to allocation during experiments and outcome assessment since our data analyses are based on objectively measurable data.

Reporting for specific materials, systems and methods

We require information from authors about some types of materials, experimental systems and methods used in many studies. Here, indicate whether each material, system or method listed is relevant to your study. If you are not sure if a list item applies to your research, read the appropriate section before selecting a response.

Materials & experimental systems

n/a	Involved in the study	
☒	☐	Antibodies
☐	☒	Eukaryotic cell lines
☒	☐	Palaeontology and archaeology
☐	☒	Animals and other organisms
☒	☐	Human research participants
☒	☐	Clinical data
☒	☐	Dual use research of concern

Methods

n/a	Involved in the study	
☒	☐	ChIP-seq
☐	☒	Flow cytometry
☒	☐	MRI-based neuroimaging

Eukaryotic cell lines

Policy information about cell lines

Cell line source(s)

4T1 cell line was purchased from Sigma-Aldrich (USA).

Authentication

4T1 cell line has been authenticated by short tandem repeat profiling.

Mycoplasma contamination

4T1 cell line was tested negative for mycoplasma contamination.

Commonly misidentified lines (See ICLAC register)

None.

Animals and other organisms

Policy information about studies involving animals; ARRIVE guidelines recommended for reporting animal research

Laboratory animals	BALB/c mice were purchased from Shanghai SLAC Laboratory Animal Co. Ltd (Shanghai, China) at the age of 5-6 weeks after birth. The female BALB/c mice were used, because the murine breast cancer model was constructed using these mice. The mice spent most of their time in the dark room environment, with light time of about 2 hours a day, environmental temperature of 26-28 degrees Celsius and humidity of 40-60%.
Wild animals	No wild animal was involved.
Field-collected samples	No field sample was collected.
Ethics oversight	All animal experiments were performed under a protocol approved by the Institutional Animal Care and Use Committee of Xiamen University.

Note that full information on the approval of the study protocol must also be provided in the manuscript.

Flow Cytometry

Plots

Confirm that:

- [x] The axis labels state the marker and fluorochrome used (e.g. CD4-FITC).
- [x] The axis scales are clearly visible. Include numbers along axes only for bottom left plot of group (a 'group' is an analysis of identical markers).
- [x] All plots are contour plots with outliers or pseudocolor plots.
- [x] A numerical value for number of cells or percentage (with statistics) is provided.

Methodology

Sample preparation	4T1 cells (200,000) were seeded into 6-well culture plates and incubated for 12 h. After treating with PBS or ITC-NPs (80 μg/mL) for 24 h, the cells were irradiated with 2 Gy of X-rays. After further incubation for 24 h, the cells were treated by trypsinization, harvesting, rinsing, and redispersing, and stained with Annexin V-FITC/PI. Then, the cell apoptosis was recorded by flow cytometer.
Instrument	Beckman Coulter Cytoflex Flow Cytometer
Software	CytExpert software
Cell population abundance	A total of 10,000 cells were analyzed per sample
Gating strategy	Gating strategies are referred to the method described in the BIO-RAD website (https://www.bio-rad-antibodies.com/flow-cytometry-gating-strategies.html). Gating was based on FSC/SCC together with fluorescent dyes and singlet populations. A forward-scatter (FSC) vs side-scatter (SSC) gate was used to gated on 4T1 cells to exclude debris. FSC-H vs FSC-A gate was used to gated on 4T1 singlet cells. The cell populations within the gate were analyzed based on the expression of markers.

- [x] Tick this box to confirm that a figure exemplifying the gating strategy is provided in the Supplementary Information.
